# Global seroprevalence of neutralizing and total antibodies against AAV vectors and their impact on the clinical gene therapy landscape: a scoping review

**DOI:** 10.3389/fmed.2026.1925844

**Published:** 2026-09-22

**Authors:** Cristina Sánchez-Gutierrez, Luisa Fernanda Bohorquez-Zambrano, Camila Garnica-Álvarez, Melvin Y. Rincon, Augusto Valderrama-Aguirre

**Affiliations:** 1 Institute for Biomedical Research (IIB), Department of Biological Sciences, Faculty of Sciences, Universidad de los Andes, Bogotá DC, Colombia; 2 Centro de Investigaciones, Fundación Cardiovascular de Colombia, Floridablanca, Colombia

**Keywords:** AAV, adeno-associated vectors, clinical trials, gene therapy, neutralizing antibodies, review, total antibodies, translational medicine

## Abstract

Pre-existing neutralizing antibodies (NAbs) and total antibodies (TAbs) against adeno-associated virus (AAV) vectors represent a major barrier to the clinical translation of AAV-based gene therapies. A comprehensive characterization of their global prevalence, determinants, and clinical implications is currently lacking. We conducted a scoping review of 50 studies published between 1968 and 2026, systematically identifying seroprevalence data across AAV serotypes, populations, and geographic regions. We complemented this evidence with a structured analysis of 222 interventional AAV gene therapy trials registered in ClinicalTrials.gov, examining serotype distribution, therapeutic areas, enrollment, and immunogenicity outcomes. We found AAV2, AAV8, and AAV5 were the most extensively studied serotypes. Seroprevalence increased consistently with age and varied by geographic region, although the limited number of studies from Africa and Latin America precludes robust regional conclusions. The median trial enrollment was 14 participants, and NAb exclusion criteria markedly reduce already limited eligible populations. Long-term follow-up data indicate that declining transgene expression is frequently associated with capsid-directed cellular immunity. Standardized serological assays, region-specific seroprevalence data, and immunomodulatory strategies are essential to maximize patient eligibility and therapeutic durability in AAV gene therapy programs globally.

## 1 Introduction

Adeno-associated virus (AAV) are a small, non-enveloped, single-stranded DNA group of viruses of the Parvoviridae family (Dependovirus genus) that are non-pathogenic to humans and require helper viruses for productive replication. In the absence of co-infection, AAV persists as episomal DNA within the host nucleus, establishing a latent state (1, 2). The icosahedral capsid, composed of VP1, VP2, and VP3, encapsidates a ∼4.7 kb genome encoding replication and capsid functions, a compact architecture that has enabled extensive vector engineering for therapeutic applications (3).

Building on these features, recombinant AAV (rAAV) vectors support efficient and durable transgene expression in both dividing and post-mitotic cells across a broad range of tissues, including the central nervous system, liver, muscle, and retina (3, 4). Distinct serotypes exhibit preferential transduction profiles, with AAV9 showing high efficiency in the central nervous system and heart (5, 6), AAV8 demonstrating broad systemic and hepatic tropism (7), and AAV5 being widely applied in retinal disorders (1, 8). Collectively, these properties have driven regulatory approval of multiple AAV-based therapies and the expansion of over 200 clinical trials targeting inherited and degenerative diseases (4, 9, 10).

rAAV are characterized by low intrinsic immunogenicity, i.e., a limited capacity to trigger innate, inflammatory responses upon vector administration and minimal pathogenicity, features that distinguish them from adenoviral, retroviral, and herpes simplex virus vectors, which frequently induce robust inflammatory responses leading to rapid elimination of transduced cells (11). This low intrinsic immunogenicity, however, does not extend to acquired humoral immunity: preexisting antibodies elicited by natural AAV exposure over the lifespan constitute a major biological constraint on AAV-mediated gene transfer, as circulating neutralizing antibodies (NAbs) directed against specific serotypes can promote rapid vector clearance prior to target tissue engagement (12). Importantly, even low NAb titers have been shown to significantly compromise transduction efficiency, with their functional impact shaped by a complex interplay between vector serotype, route of administration, target organ, and host serostatus, thereby representing a particularly significant challenge for systemically administered therapies (13).

Despite limited activation of innate immune pathways following AAV exposure, potent adaptive immune responses have been documented against both wild-type and rAAV, resulting in durable immunological memory directed toward capsid epitopes and, in some contexts, the expressed transgene product (11). The magnitude and quality of these responses are strongly influenced by serotype-specific tropism, as certain capsids are capable of transducing antigen-presenting cells, thus amplifying antigen presentation and enhancing immunogenicity, particularly in the context of heterologous or immunologically novel transgene expression (14). Notably, localized administration to immune-privileged anatomical sites, including the eye (15, 16) and central nervous system, is comparatively less susceptible to antibody-mediated neutralization, underscoring the critical role of vector design and delivery strategy in modulating host immune responses (17).

At the population level, anti-AAV antibody seroprevalence follows a dynamic, age-dependent trajectory, characterized by high titers in early life due to passive maternal transfer, a decline during late infancy, and a progressive increase throughout adulthood driven by cumulative exposure to wild-type AAV (18–20). Reported prevalence varies considerably across serotypes and is further modulated by demographic factors, geographic region, underlying health status, and assay methodology (12, 21, 22). These observations underscore the importance of accurately defining serotype-specific antibody prevalence and titers to inform patient stratification and support rational vector selection.

Given the profound impact of preexisting immunity on AAV-mediated gene transfer, systematic assessment of anti-AAV seroprevalence has become a critical component of patient stratification in clinical gene therapy (14). Current screening strategies primarily rely on the detection of either total antibodies (TAbs), which reflect prior exposure and broad humoral reactivity to the AAV capsid, or neutralizing antibodies (NAbs), which directly interfere with vector transduction and represent the most reliable predictor of therapeutic efficacy (23).

Here, we conduct a scoping review to map the global evidence on the reported prevalence of total antibodies (TAbs) and neutralizing antibodies (NAbs) against adeno-associated virus (AAV) vectors in the general population and selected subpopulations, and complement this evidence with a systematic characterization of the current AAV gene therapy clinical trial landscape retrieved from ClinicalTrials.gov, in order to contextualize seroprevalence data within the therapeutic settings where pre-existing immunity poses the greatest translational challenge. By summarizing data across AAV serotypes, populations, and assay approaches, and synthesizing the underlying immune mechanisms associated with each serotype, this review aims to highlight patterns, variability, and key gaps in the current literature that are relevant to the translational application of AAV-based gene therapies.

## 2 Methodology

### 2.1 Scoping review

#### 2.1.1 Study design

This scoping review was conducted in accordance with the Preferred Reporting Items for Systematic Reviews and Meta-Analyses extension for Scoping Reviews (PRISMA-ScR) guidelines (24). The review process followed the recommended framework, including identification, screening, eligibility assessment, and inclusion of studies.

#### 2.1.2 Information sources and search strategy

A structured search strategy was designed to identify peer-reviewed articles reporting data on anti-AAV antibodies. Searches were conducted across three electronic databases PubMed, ScienceDirect, and Scopus covering studies published in English from database inception through March 2026, with no date of publication restriction. The following Boolean keyword string was applied consistently across all three databases: (AAV and “adeno-associated virus”) or (“viral vector”) AND (“antibodies” OR “total antibodies”) AND (“neutralizing antibodies”) AND (“prevalence”) AND (“gene therapy”).

All search terms were verified against Medical Subject Headings (MeSH) controlled vocabulary prior to execution. In PubMed, MeSH terms were applied in combination with free-text keywords to maximize retrieval sensitivity; in Scopus and ScienceDirect, equivalent free-text terms were searched across title, abstract, and keyword fields. Records retrieved from the three databases were consolidated and duplicate entries removed prior to screening. Reference lists of all included studies were additionally screened to identify eligible publications not captured by the electronic searches (backward citation tracking). This search strategy favored specificity over sensitivity: studies using only the abbreviation “AAV” without the spelled-out term “adeno-associated virus,” or reporting IgG data without explicitly distinguishing between total and neutralizing antibodies, may not have been captured. The backward citation tracking step described above partially mitigates this risk, as it was designed to identify eligible publications missed by the electronic search strategy, although it cannot fully compensate for the lower sensitivity of the initial search

#### 2.1.3 Eligibility criteria

Studies were included if they met all of the following criteria: (i) original research articles reporting primary empirical data collected directly from human participants, including cross-sectional surveys, cohort studies, and observational studies; (ii) reporting quantitative seroprevalence data for NAbs, either alone or together with TAbs, against at least one AAV serotype; and (iii) conducted in human populations, regardless of age group, clinical condition, or geographic region.

Studies were excluded if they: (i) were secondary literature, including review articles, systematic reviews, meta-analyses, editorials, commentaries, or clinical practice guidelines; (ii) were clinical trial reports primarily reporting therapeutic efficacy or safety outcomes without independent seroprevalence data; (iii) reported data exclusively from animal models or *in vitro* systems without a human component; (iv) did not provide quantitative seroprevalence estimates, defined as the proportion of NAb-positive or TAb-positive individuals within a defined population;(v) focused exclusively on capsid engineering, vector development, or therapeutic optimization without reporting population-level immunological prevalence data; or (vi) reported TAb data exclusively, without accompanying NAb data.

#### 2.1.4 Study selection

Retrieved records were screened in two sequential stages. In the first stage, titles and abstracts were independently reviewed by two authors to exclude clearly irrelevant studies based on the predefined eligibility criteria. In the second stage, full-text articles of potentially eligible records were assessed for inclusion. Disagreements at either stage were resolved through discussion and consensus with a third reviewer. The complete study selection process is reported in accordance with PRISMA-ScR guidelines and summarized in Figure 1.

**Figure 1:**
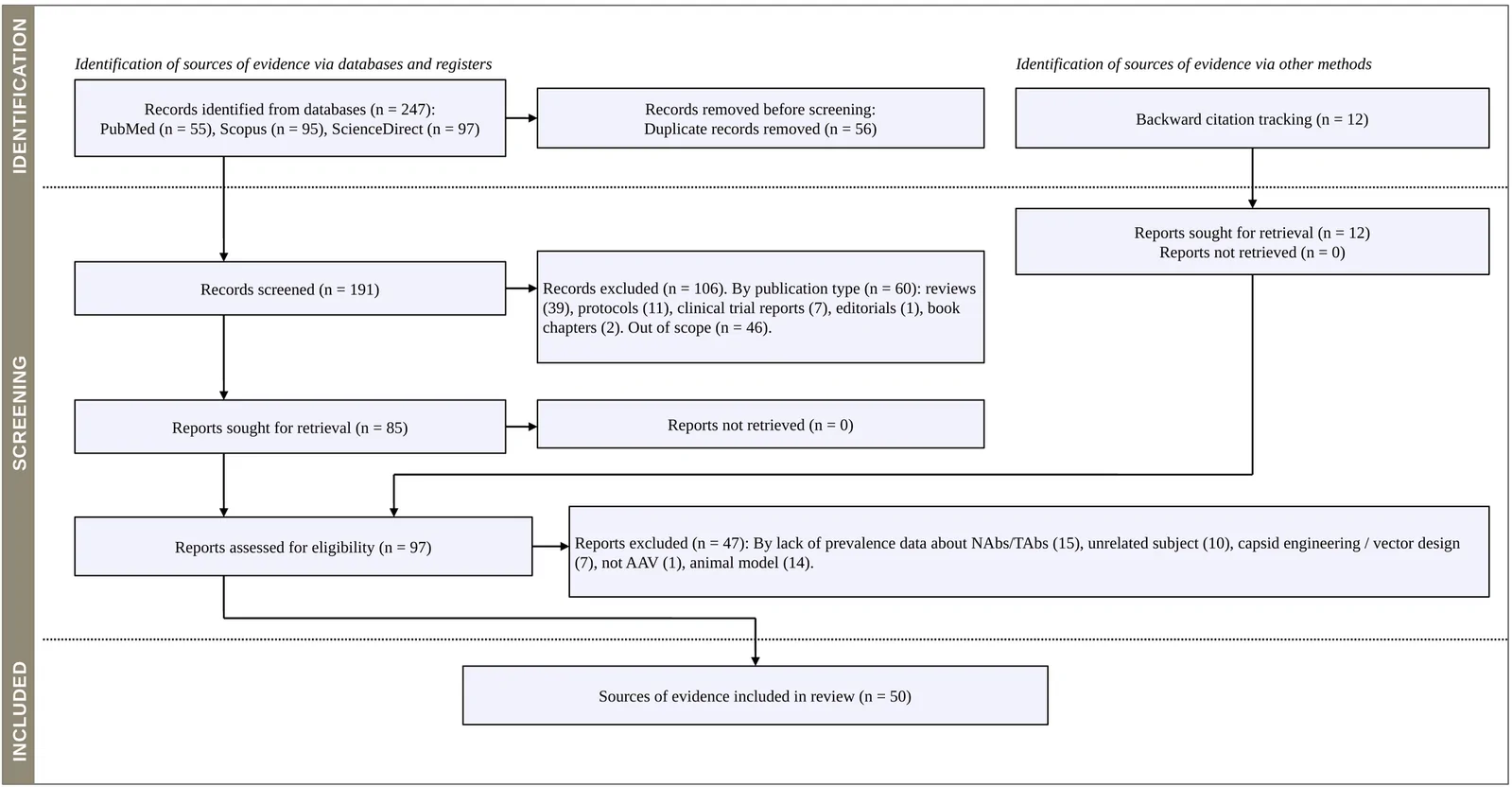
PRISMA-ScR flow diagram of the study identification, screening, and inclusion process. Eligible records reported quantitative seroprevalence data for neutralizing antibodies (NAbs) alone or in combination with total antibodies (TAbs); records reporting TAb data exclusively, in the absence of NAb data, did not meet the eligibility criteria and were excluded at the full-text screening stage.

#### 2.1.5 Data extraction and charting

Data extraction was performed independently by three reviewers using a standardized data collection form. Extracted data were cross-checked across reviewers, and any discrepancies were resolved through discussion and consensus. The following variables were extracted from each included study: study design and publication year; country of origin and study population characteristics (sample size, age group, health status); AAV serotype(s) evaluated; type of antibody assessed (NAbs, TAbs, or both); reported seroprevalence values and, where available, antibody titer data; and methodological characteristics of the assay used, including platform type, serum dilution scheme, and seropositivity cutoff.

#### 2.1.6 Data synthesis

Data synthesis was conducted using a descriptive approach consistent with scoping review methodology. Quantitative data were summarized as counts and percentages of studies, and as means where applicable. Data management and visualization were performed using RStudio (version 2024.12.1 + 563), and findings were organized into summary tables and figures mapping the distribution of studies across serotypes, geographic regions, and study populations. In accordance with established scoping review methodology (24), no formal critical appraisal of individual studies was performed, as the objective was to map and characterize the available evidence rather than evaluate methodological quality; inferential statistical analyses were similarly not conducted. This contrasts with systematic reviews, for which formal risk-of-bias appraisal is required as a basis for evidence synthesis and recommendations; the methodological heterogeneity noted throughout the Results (assay platform, dilution scheme, and seropositivity cutoff) is reported descriptively for this reason.

### 2.2 Complementary analysis of registered AAV clinical trials

To complement the seroprevalence evidence with a contextual overview of the current clinical landscape, a structured search was conducted on the ClinicalTrials.gov registry (data cutoff: March 31, 2026) to identify registered interventional studies evaluating rAAV-based gene therapy in human populations. The search was performed using the term ‘Gene therapy AAV’ in the ‘Other terms’ field, retrieving 260 registered records. All records were individually verified through NCT-level lookup to confirm the nature of the intervention and the appropriateness of the ‘AAV’ acronym (i.e., adeno-associated virus rather than ANCA-associated vasculitis); a record was confirmed for inclusion when it explicitly described an rAAV-based gene therapy intervention administered to human participants. This analysis is intended as a descriptive, complementary characterization of the registered trial landscape rather than an exhaustive global registry review; trials registered exclusively in other databases (e.g., EU Clinical Trials Register, Chinese Clinical Trial Registry) may not be fully represented.

#### 2.2.1 Inclusion and exclusion criteria

Records were included in the primary analytical dataset if they met all of the following criteria: (1) use of a rAAV as the principal gene delivery vehicle administered directly to human participants; (2) interventional study design (Early Phase 1 through Phase 3); and (3) designation of AAV gene therapy as the primary intervention, rather than a preparatory, screening, or natural history protocol. A total of 11 records were excluded for the following reasons: two were flagged during manual review as observational designs with no AAV vector administration, and one as a CAR-T–based therapy; three corresponded to natural history studies or registries where ‘AAV’ referred to ANCA-associated vasculitis; and five corresponded to non-AAV interventions or non-interventional records, including lentiviral vector trials, CAR-T therapies, and biobank registries. Following exclusion, 249 records were retained. Of these, 222 met full inclusion criteria as interventional trials and constitute the primary analytical dataset; the remaining 27 comprising long-term follow-up extensions, NAb/TAb pre-screening studies, and one expanded-access single-patient IND were classified as observational/reference records.

#### 2.2.2 Data extraction and classification

Data extraction was conducted using a predefined framework to ensure consistency and reproducibility across all eligible interventional studies. For each record, the following variables were systematically extracted: NCT identifier, study title, recruitment status, clinical phase, study design, target condition, therapeutic area, intervention characteristics, AAV serotype, sponsor, sex eligibility, age category (pediatric, adult, or mixed), estimated enrollment, funding source, geographic location of the primary study site, availability of reported results, and study start and completion dates. Individual trial records discussed in the text are identified by their unique NCT registry number; the complete dataset of 222 interventional trials, including all extracted variables, is provided in Supplementary Information 2.

AAV serotypes were identified through structured text mining of intervention descriptions and study titles using regular expression–based pattern matching, enabling consistent annotation of both canonical and engineered AAV variants. Extracted serotype annotations were individually confirmed against the corresponding registry record to ensure accuracy. Disease indications were categorized into predefined therapeutic areas using a hierarchical keyword-based classification scheme developed *a priori* by the research team and applied consistently across all records, allowing standardized grouping of related conditions while preserving disease-level specificity.

Records not meeting full inclusion criteria but considered relevant for contextual interpretation such as long-term follow-up studies, antibody pre-screening protocols (NAbs and TAbs), and expanded-access cases were retained as a secondary reference dataset. Although not included in the primary analytical dataset, these records were used to support the contextual interpretation of immunogenicity profiles, long-term outcomes, and seroprevalence-related variables.

## 3 Results

### 3.1 Scoping review

A total of 247 records were identified through database searching: PubMed (*n* = 55), ScienceDirect (*n* = 97), and Scopus (*n* = 95). After removing 56 duplicate records, 191 unique records were screened by title and abstract. Of these, 106 records were excluded: 60 based on publication type including review articles, editorials, clinical trial reports, methods and protocol papers, and book chapters and 46 as clearly outside the scope of the review, primarily studies limited exclusively to animal models, studies not reporting quantitative antibody data, or studies focused on non-AAV viral vectors. A total of 85 records proceeded to full-text assessment. Clinical trial reports were excluded at this stage; as interventional trial data were analyzed separately in Section 3.2. An additional 12 records were identified through backward citation tracking of included studies (12, 25–27) yielding 97 records assessed for full-text eligibility. Following full-text review, 47 records were excluded for the following reasons: studies conducted exclusively in animal models; studies not reporting NAb prevalence data; studies lacking quantitative seroprevalence estimates; studies evaluating TAbs only without NAb data, including one record from backward citation tracking; one additional record from backward citation tracking with insufficient NAb prevalence data; and studies focused solely on capsid engineering without reporting population-level immunological outcomes. Ultimately, 50 original research articles met all inclusion criteria and were included in the scoping review (Figure 1).

#### 3.1.1 Distribution of research articles by year

The 50 included research articles reporting on NAbs and/or TAbs against AAV spanned publications from 1968 through March 31, 2026. Early reports were sporadic, with isolated publications in 1968 and 1999, followed by a gradual increase from the mid-2000s and a marked acceleration after 2017. The highest annual output was observed in 2019 (*n* = 9; 18% of the total), followed by 2022 (*n* = 7; 14%) (Figure 2). Notably, the same period shows a parallel and larger increase in the number of AAV gene therapy clinical trials initiated per year, particularly from 2022 onward; this trend is examined in detail in Section 3.2, which characterizes the clinical trial landscape.

**Figure 2:**
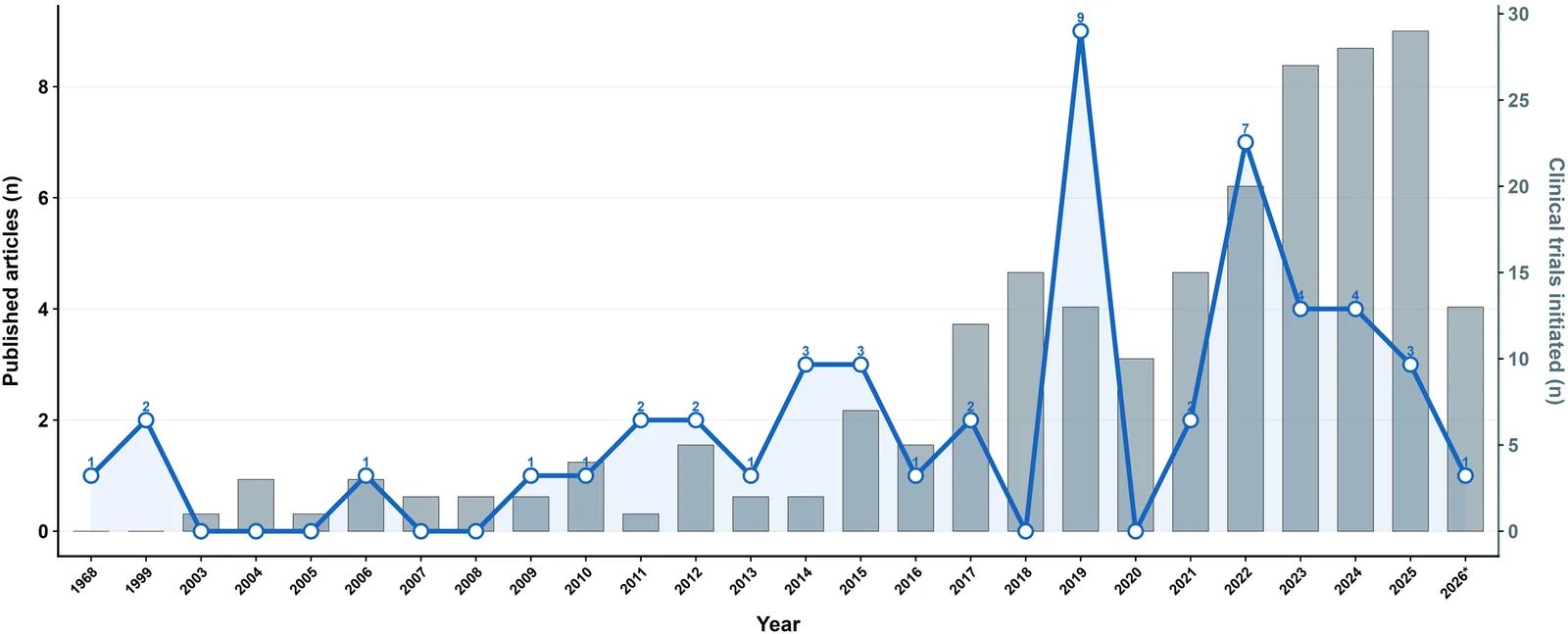
Temporal trend of AAV antibody seroprevalence research and clinical trial activity, 1968–2026. The blue line and points indicate the number of peer-reviewed articles reporting NAb data, alone or in combination with TAb data, published per year (left axis; *n* = 50 total). Grey bars indicate the number of AAV gene therapy clinical trials with a registered start date in ClinicalTrials.gov for the corresponding year (right axis). Data for 2026 (marked with an asterisk) are partial, covering records identified through March 31, 2026, only.

#### 3.1.2 Distribution of research articles by serotypes/variants

In total, the scoping review included studies evaluating ten AAV serotypes (AAV1–AAV9 and AAV12) and several engineered variants, including AAV3B (Figure 3). Most serotype-evaluation instances (*n* = 195 total NAb/TAb assessments across all serotypes and variants) focused on AAV2 (21.5%), AAV8 (17.4%), AAV5 (14.9%), and AAV9 (12.3%). NAbs against AAV2 were assessed in 32 studies, of which 10 also evaluated TAbs. For AAV8, 25 studies analyzed NAbs and 9 included TAbs. AAV5 was examined in 22 studies, with 7 also reporting TAbs. Finally, 18 studies investigated NAbs against AAV9, including 6 that quantified TAbs (Figure 3).

**Figure 3:**
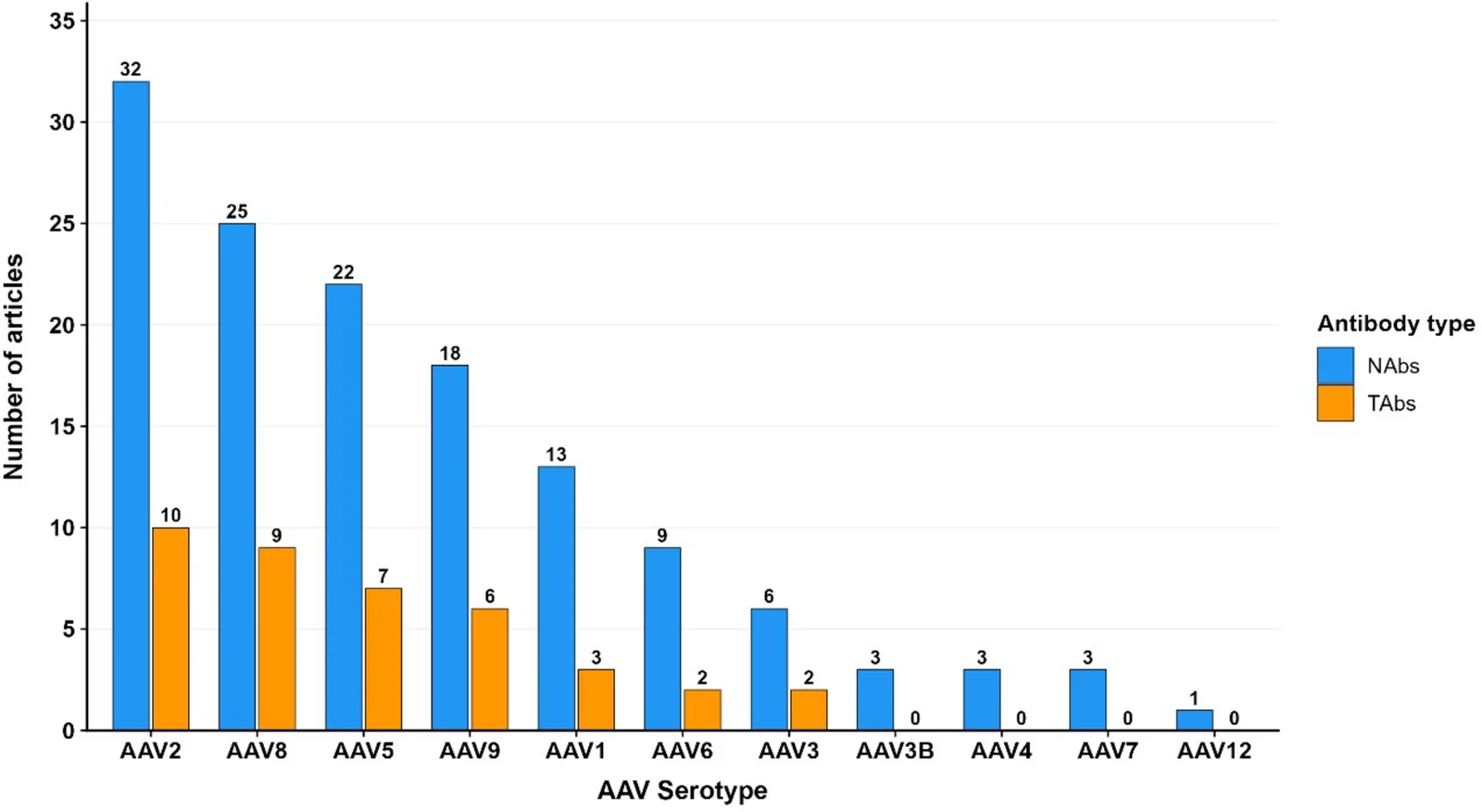
Number of articles evaluating NAbs and TAbs against AAV serotypes among the 50 included studies. As a single study could evaluate multiple serotypes, bars represent the number of serotype-specific evaluations rather than unique studies. AAV2 was the most frequently examined, followed by AAV8 and AAV5, whereas AAV12 was the least frequently studied among the serotypes shown.

Among AAV variants, AAV3B was the most frequently evaluated, with 3 NAb assessments and no TAb data (1.5%; Figure 3). The remaining engineered variants including AAVrh.10 (2 NAb and 1 TAb assessments), AAV2.GL, AAV2.NN, AAVrh74, AAVLK03, and AAVWT (each reported in 1–2 studies), and AAAV, AAV2.5, AAV44.9, AAVrh32.33, AAVRh74var, AAVzk2, BAAV, and BNP116 (each reported in a single study) were not individually plotted in Figure 3 due to their low frequency. Overall, the characterization of AAV variants remained limited compared to that of the naturally occurring serotypes shown in Figure 3.

#### 3.1.3 Distribution of research articles by geographic region

Seroprevalence data were reported from 50 studies encompassing 32 countries across five continents (Figure 4, see Supplementary Information 1). The global evidence base displayed a marked geographic asymmetry, with research activity mainly concentrated in high-income regions. North America and Europe collectively accounted for the majority of reports, led by the United States (*n* = 21; 42%), Germany (*n* = 10; 20%), and France (*n* = 7; 14%). Within Europe, study representation was broadly distributed across Western and Central countries, while in Asia, data were predominantly generated from China and Japan, reflecting the regional leadership of these nations in AAV vector research. In contrast, contributions from Oceania, Latin America, and Africa remained limited, together constituting approximately 22% of total studies. Given the very limited number of studies from these regions, any inferences regarding regional seroprevalence trends in Africa and Latin America should be interpreted with caution, as the available data are insufficient to support robust geographic conclusions.

**Figure 4:**
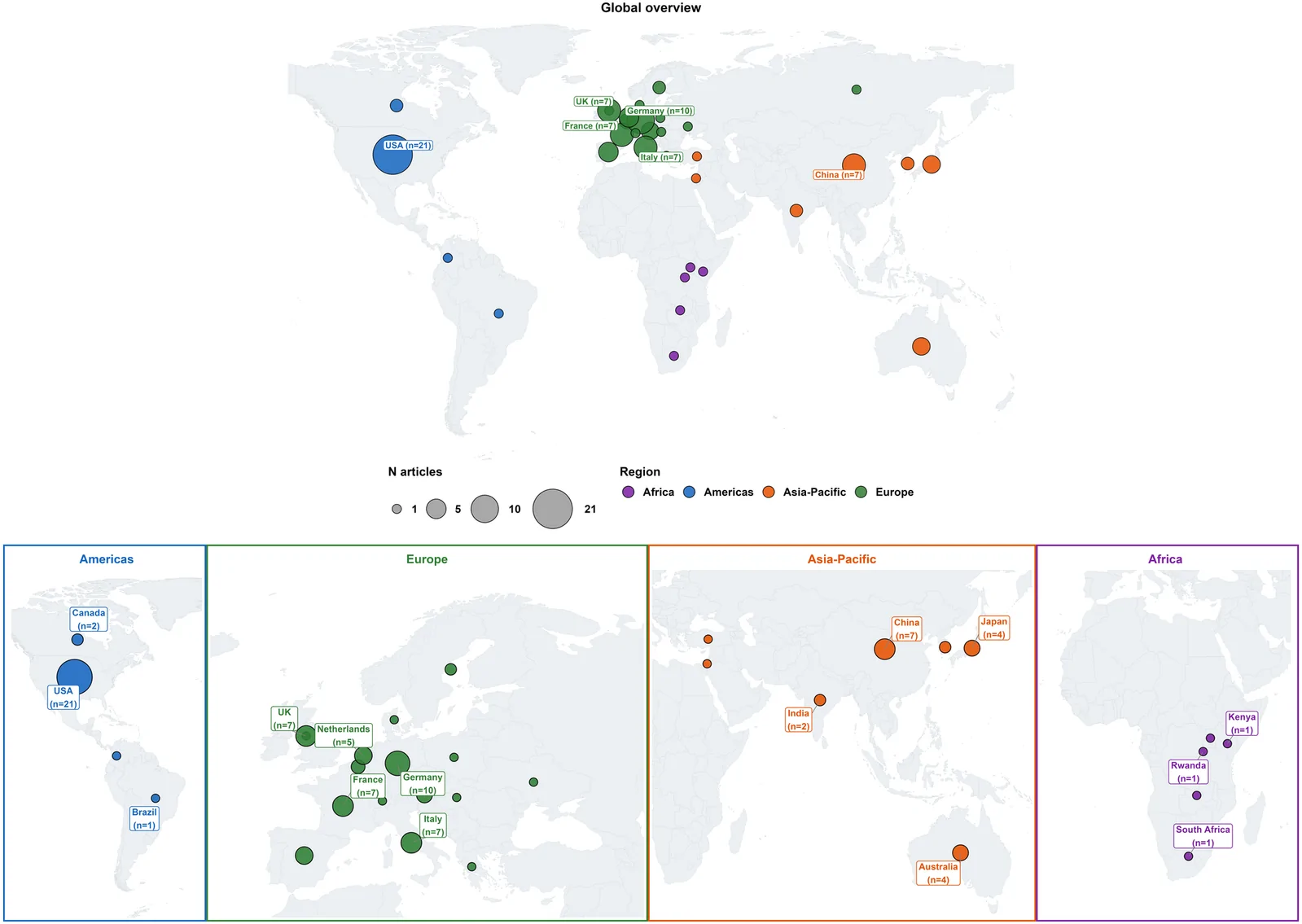
Geographic distribution of studies included in the scoping review (*n* = 50). The global overview map (top) displays each contributing country as a proportional circle sized by the number of articles, color-coded by region. Regional inset maps (bottom) show country-level detail within the Americas, Europe, Asia-Pacific, and Africa; only the most representative countries per region are labeled by name, while all countries are shown as proportional circles regardless of label.

#### 3.1.4 Distribution of research articles by health condition

Most research articles examining anti-AAV TAbs and NAbs were conducted in healthy individuals (*n* = 33; 66%), providing baseline seroepidemiological references for population-level comparisons; a smaller number of studies recruited laboratory personnel (*n* = 1), together comprising 34 studies (68%) within the broader “Other/Controls” category (Figure 5).

**Figure 5:**
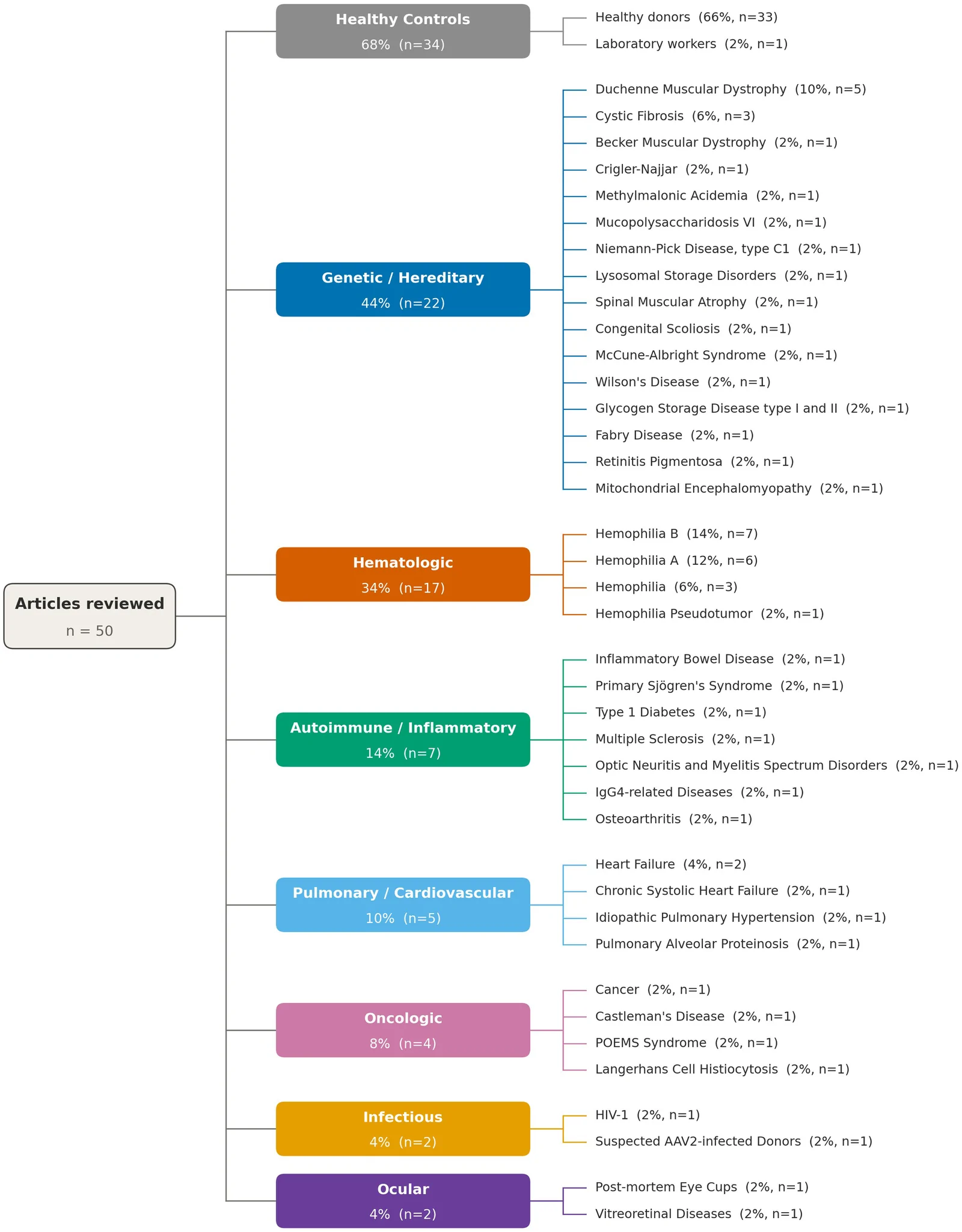
Hierarchical tree diagram classifying the 50 reviewed articles across eight health condition categories and their constituent conditions. Each category box reports the proportion and number of articles (e.g., Healthy Controls, 68%, *n* = 34); individual conditions are listed with their corresponding values and connected to their parent category by a line. As articles could be assigned to more than one category, category-level percentages sum to more than 100%; within each category, condition-level counts sum exactly to the category total (e.g., the 16 conditions under Genetic/Hereditary sum to 44%, *n* = 22). Both the Genetic/Hereditary and Autoimmune/Inflammatory categories display a long-tail distribution, with most constituent conditions represented by a single article (*n* = 1; 2%).

Among disease-associated populations, genetic and hereditary disorders constituted the second most frequently analyzed category overall (*n* = 22; 44%), encompassing Duchenne (*n* = 5) and Becker (*n* = 1) muscular dystrophies, cystic fibrosis (*n* = 3), and a range of rare metabolic and lysosomal storage diseases each represented by a single study, underscoring the clinical translation of AAV vectors in monogenic disorders. Hematologic conditions represented the third most frequent category (*n* = 17; 34%), driven primarily by hemophilia A (*n* = 6; 12%) and hemophilia B (*n* = 7; 14%) showing the established role of hemophilia as a leading clinical model for AAV-mediated gene therapy, with an additional 4 studies describing hemophilia without subtype distinction or hemophilia-associated pseudotumor.

In contrast, autoimmune/inflammatory conditions were the fourth most represented category (*n* = 7; 14%), encompassing inflammatory bowel disease, primary Sjögren's syndrome, type 1 diabetes, multiple sclerosis, optic neuritis and myelitis spectrum disorders, IgG4-related diseases, and osteoarthritis. The inclusion of osteoarthritis in this category reflects its status as the target indication of an active AAV2.5-based intra-articular gene therapy program, in which pre-existing neutralizing antibodies in synovial fluid represent a direct translational barrier. Pulmonary/cardiovascular (*n* = 5; 10%), oncologic (*n* = 4; 8%), infectious (*n* = 2; 4%), and ocular (*n* = 2; 4%) conditions were less commonly assessed, generally within exploratory or secondary study contexts (see Supplementary Information 1). Overall, the distribution of study populations reflects a predominant emphasis on monogenic and hematologic diseases, consistent with the leading therapeutic priorities driving current AAV gene transfer research.

#### 3.1.5 Prevalence of TAbs and NAbs across research articles

Across the reviewed studies, TAbs were evaluated in less than half of the reports (19/50), contrasting with the universal assessment of NAbs. Because included studies employed heterogeneous assay platforms, reporter systems, serum dilution schemes, and seropositivity cutoffs (Supplementary Table S1), the prevalence values summarized below are presented descriptively and are not directly comparable across studies; observed ranges reflect this methodological heterogeneity rather than necessarily indicating true biological variation.

Taking this into account, AAV2 showed the highest reported TAb prevalence values among the serotypes assessed, with individual studies reporting values often exceeding 70% and reaching 90%–96% in healthy donors and individuals with cystic fibrosis (15, 28). Reported prevalence for AAV8 and AAV3 was more variable across cohorts (12, 29–31), typically ranging from 25%–45%, with one Indian cohort reporting values exceeding 90% for AAV3 (26, 32). In contrast, AAV5 and the engineered variant AAV-LK03 showed comparatively lower IgG detection rates, often below 50% (8, 14). Given the assay heterogeneity noted above, this overall pattern supports, but does not formally confirm, the immunodominance of AAV2 reported elsewhere in the literature, possibly reflecting its natural tropism and widespread environmental exposure. Cross-study comparisons of these ranges should therefore be interpreted with caution and not as evidence of geographic or demographic trends.

As with the TAb data above, the following NAb prevalence values derive from assays with differing cutoffs and dilution schemes and are presented descriptively rather than as directly comparable estimates (Supplementary Table S1). Across the 50 studies analyzed, AAV2 was the serotype most frequently associated with high reported prevalence, with individual values ranging from 22.1% in healthy donors (18) to 98% in patients with vitreoretinal diseases in South Korea (16). High prevalence values were also described in healthy and HIV-1-infected individuals (96%) (33), Spanish donors (71%), and Chinese donors (92%) (34), while intermediate values were reported in DMD patients and healthy donors (56%–64%) (2) and in Colombian populations (25, 35–37).

AAV8 and AAV1 followed in prevalence, reported in 8/50 and 5/50 studies, respectively. AAV8 reached 94% in Dutch healthy donors (38) and 42% in MPS VI patients (39), whereas AAV1 ranged from 39.7–74.9%, including 58.8% in French donors (40) and approximately 60% in European and U.S. heart failure cohorts (41). Other serotypes were less frequent. AAV4 seroprevalence varied from 0% (42) to 44% (38), AAV5 ranged from less than 5% to 40.2%, and AAVrh32.33 remained rare (under 2%) (21). AAV9 showed heterogeneous detection (0–57.8%), including 27.4% in Japanese cohorts (43) and 36–40.9% in DMD and Niemann-Pick C1 patients (44). Minor variants such as AAV12 (0%–8%), AAV44.9 (27%), and AAVzk2 (0%) were sporadically reported (35, 36, 45) (see Supplementary Information 1).

### 3.2 Clinical trials using AAV gene therapy

A systematic search of the ClinicalTrials.gov registry identified 260 registered records involving AAV gene therapy. After applying the eligibility criteria described in the methods section, 11 records were excluded: 3 observational studies or CAR-T-based trials identified during manual review; 3 natural history studies or entries where the acronym ‘AAV’ referred to ANCA-associated vasculitis; and 5 records corresponding to non-AAV modalities (lentiviral vectors, CAR-T therapies, or biobanks). The final dataset comprised 249 records: 222 interventional trials constituting the primary analytical dataset, and 27 observational or long-term follow-up studies retained as a reference category to support the interpretation of immune response outcomes and pre-existing antibody seroprevalence. Unless otherwise specified, all counts and percentages reported in this section refer to the 222 interventional trials in the primary analytical dataset.

#### 3.2.1 Clinical phases

The phase distribution reflects a pipeline still concentrated in early development stages. The largest single group consisted of Phase 1/2 combined designs (*n* = 94, 42.3%), followed by Phase 1 (*n* = 53, 23.9%), Early Phase 1 (*n* = 24, 10.8%), Phase 3 (*n* = 18, 8.1%), Phase 2 (*n* = 17, 7.7%), Phase 2/3 (*n* = 6, 2.7%), and trials without a specified phase (*n* = 10, 4.5%) (Figure 6). Of the 18 Phase 3 trials, 8 (44.4%) had posted results in the registry, representing the primary source of high-quality efficacy and immunogenicity data available for the most advanced AAV programs.

**Figure 6:**
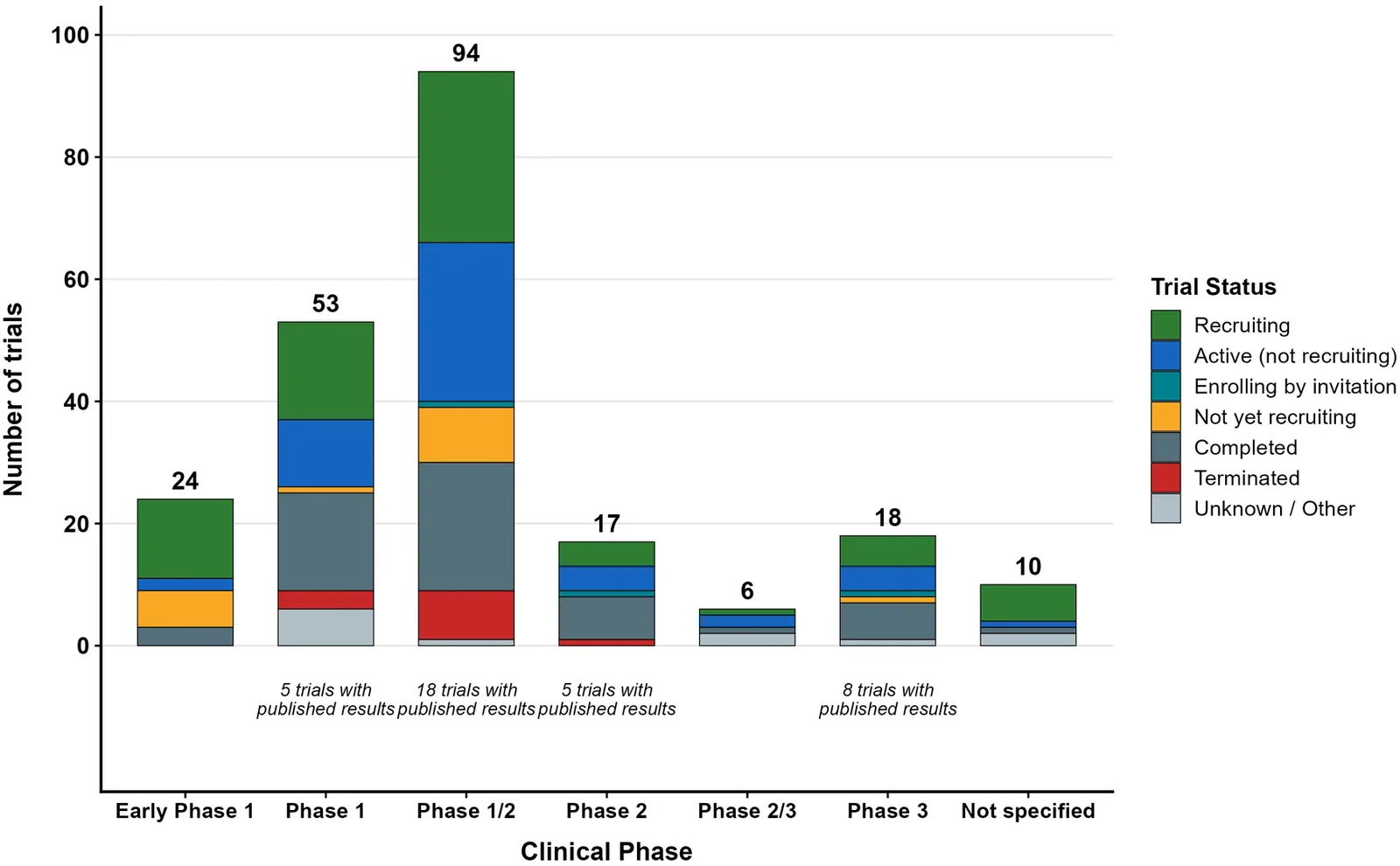
Distribution of AAV gene therapy clinical trials by phase and recruitment status (*n* = 222 total interventional trials). Stacked bars show the number of trials per phase, colored by trial status. Total counts per phase are indicated above each bar; the number of trials with posted results within each phase is shown below the *x*-axis.

#### 3.2.2 Therapeutic areas and target diseases

The 222 interventional trials addressed 11 broad therapeutic areas. Ophthalmology was the most represented category (*n* = 61, 27.5%), reflecting the ocular immune privilege and the relatively accessible anatomy of the eye that have historically made it a preferred target for AAV gene delivery. The most frequently studied ophthalmic conditions included retinitis pigmentosa (RP), neovascular age-related macular degeneration (nAMD), choroideremia, achromatopsia, X-linked retinoschisis, and Leber congenital amaurosis (LCA), with representative trials including NCT04611503 (RP, Phase 1/2), NCT07482176 and NCT06856577 (nAMD, Phase 3), NCT03496012 (choroideremia, Phase 3), NCT03001310 and NCT03758404 (achromatopsia, Phase 1/2), NCT02317887 (X-linked retinoschisis, Phase 1/2), and NCT00999609 (LCA, Phase 3).

A further 46 trials (20.7%) could not be assigned to a specific disease category based on available registry information and were grouped as “Other,” representing the second largest category in the dataset. Hematological disorders represented 30 trials (13.5%), with hemophilia A and hemophilia B collectively representing the most frequent individual diagnoses across the entire dataset. Neurological conditions comprised 27 trials (12.2%), with Parkinson's disease, Huntington's disease, Rett syndrome, and Canavan disease among the most frequent targets. Neuromuscular diseases constituted 22 trials (9.9%), predominantly Duchenne muscular dystrophy (DMD) and late-onset Pompe disease. Metabolic and lysosomal storage disorders represented 15 trials (6.8%), followed by hepatic indications (*n* = 13, 5.9%), which included Crigler-Najjar syndrome (NCT03466463, NCT06518005), ornithine transcarbamylase (OTC) deficiency (NCT02991144, NCT06255782), alpha-1 antitrypsin deficiency (NCT01054339), Wilson's disease (NCT04884815, NCT04537377), and phenylketonuria (NCT04480567). Oncology (*n* = 3), pulmonary (*n* = 2), cardiovascular (*n* = 2), and hearing/audiology (*n* = 1) accounted for the remaining trials (Figure 7).

**Figure 7:**
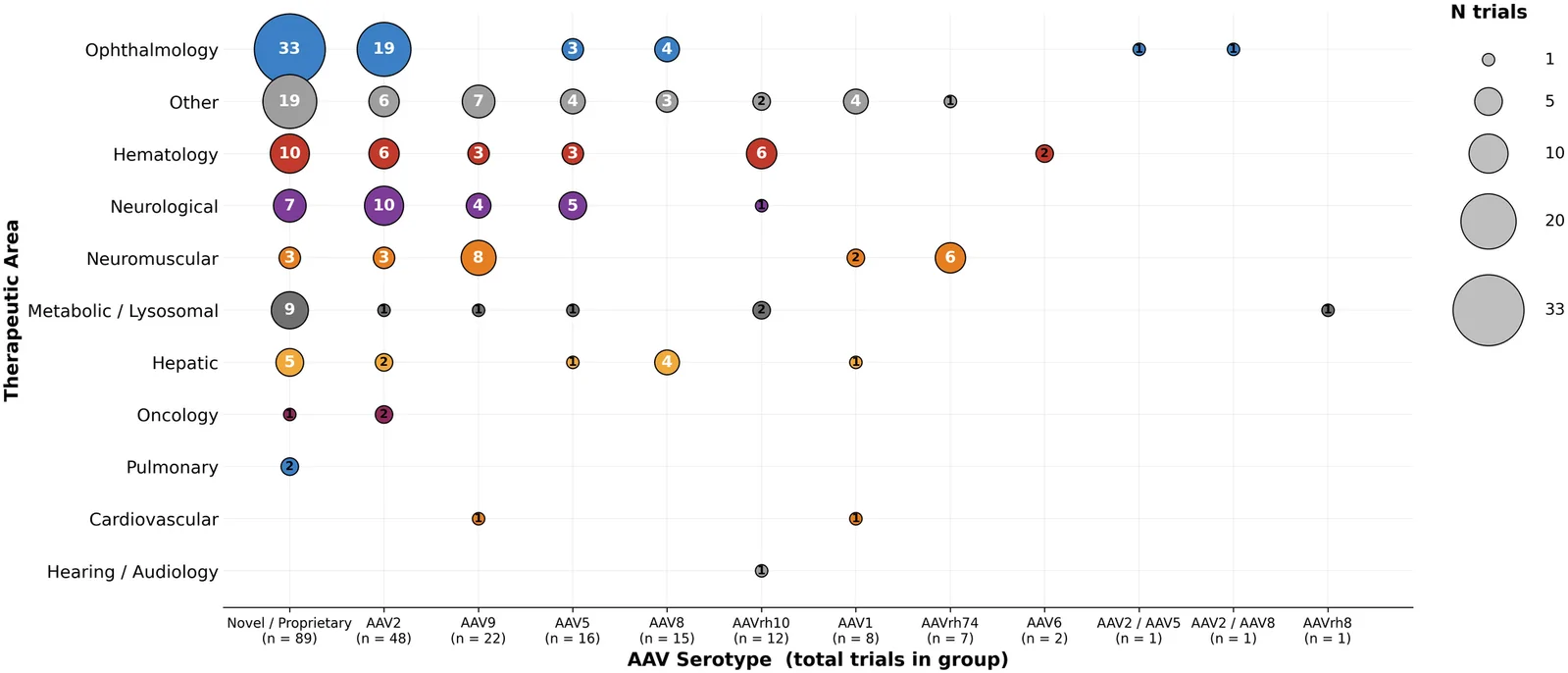
Distribution of clinical trials by AAV serotype and therapeutic area (*n* = 222). Bubble size and inset numbers indicate trial counts; total trials per serotype group are shown in parentheses on the *x*-axis. “Novel/Proprietary” (*n* = 89) denotes engineered capsids and platform-specific vectors without a disclosed canonical serotype (e.g., Spark Therapeutics SPK-series, 4D Molecular Therapeutics 4D-series, PHP-eB variants). Dual-serotype hybrid capsids (AAV2/AAV5, AAV2/AAV8, AAVrh8 dual-vector) are each shown individually (*n* = 1).

#### 3.2.3 AAV serotype distribution

Serotype information was extracted from intervention descriptions, study titles, and brief summaries, and was supplemented by published literature and patent databases where registry entries lacked sufficient detail for classification. The largest proportion of trials (*n* = 89, 40.1%) employed engineered, proprietary, or undisclosed capsids classified as Novel/Proprietary, including 4D-series (4D-C102, 4D-R100), Spark-derived variants (Spark100, SPK-series), FLT capsids (Freeline Therapeutics), and the AAV.44LOOP platform (Adverum Biotechnologies), among others. Among trials with a well-established serotype, AAV2 was the most frequently deployed (*n* = 48, 21.6%), consistent with its historical precedence in ophthalmological and neurological applications and its role as the reference serotype in the majority of published NAb seroprevalence studies. AAV9 was the second most common serotype (*n* = 21, 9.5%), followed by AAV5 (*n* = 16, 7.2%), AAV8 (*n* = 15, 6.8%), AAVrh10 (*n* = 12, 5.4%), AAV1 (*n* = 8, 3.6%), AAVrh74 (*n* = 7, 3.2%), and AAV6 (*n* = 2, 0.9%) (Figure 7).

Three additional trials employed alternative capsid or formulation strategies: two utilized pseudotyped vectors, including an AAV2/5 construct (NCT02781480, LCA) and an AAV2/8 construct (NCT03758404, achromatopsia), while a third implemented an AAVrh.8 dual-vector strategy (NCT04669535, Tay-Sachs/Sandhoff disease). Three further trials used standard serotypes or basic variants within their established categories rather than engineered or dual-vector systems: an AAV2-based construct delivered via the hepatic artery (NCT00076557, hemophilia B), a single AAV9 vector (NCT02716246 and NCT04088734, MPS III), and an AAV8 vector encoding a broadly neutralizing antibody (NCT03374202, HIV infection).The predominance of AAV2, AAV5, AAV8, and AAV9 in the clinical landscape directly corresponds to the serotypes most extensively evaluated in NAb and TAb seroprevalence studies, as detailed in subsequent sections of this review.

#### 3.2.4 Number of trials, recruitment status, and enrollment

Among the 222 interventional trials, 73 (32.9%) were actively recruiting at the time of data extraction, 55 (24.8%) had been completed, and 50 (22.5%) were active but no longer recruiting. Seventeen trials (7.7%) had not yet initiated recruitment, 12 (5.4%) had been terminated, 11 (5.0%) reported an unknown status, 3 (1.4%) were enrolling by invitation only, and 1 (0.5%) had been formally withdrawn (NCT06297486) prior to initiating recruitment. Of all 222 trials, 36 (16.2%) had posted results in the registry. Terminated trials covered a heterogeneous set of indications including hemophilia A (NCT03520712), hemophilia B (NCT00076557, NCT01620801, NCT02618915), Parkinson's disease (NCT00643890, NCT02418598), Fabry disease (NCT04040049), Duchenne muscular dystrophy (NCT03362502), GM1 gangliosidosis (NCT04273269), Tay-Sachs and Sandhoff disease (NCT04669535), retinitis pigmentosa (NCT03328130), and homozygous familial hypercholesterolemia (NCT02651675), showing the range of challenges encountered in early-phase AAV development including immune-mediated hepatotoxicity, insufficient efficacy, manufacturing failures, and commercial decisions.

Enrollment across the 222 trials ranged from 0 to 284 participants per trial, with a median of 14 participants, a mean of 26.5, and an estimated total of 5,884 participants across all studies. The single trial with zero enrollment (NCT06297486, hemophilia A, SPK-8011; Spark Therapeutics) corresponds to a Phase 3 study that was formally withdrawn prior to initiating recruitment and therefore contributes no participants to the cumulative total. The two trials with the largest enrollment (*n* = 284 each) were Phase 3 studies of ixoberogene soroparvovec for neovascular AMD (NCT07482176 and NCT06856577), a condition with higher population prevalence than the rare monogenic diseases that constitute the majority of AAV trial indications. The consistently small sample sizes across this dataset have direct implications for NAb-based patient eligibility: applying standard NAb exclusion thresholds to an already limited eligible population can reduce the available trial cohort to critically low numbers when a considerable proportion of the target population harbors pre-existing neutralizing antibodies against the vector serotype in use.

#### 3.2.5 Geographic distribution and sponsorship

Trials were geographically concentrated in North America and Asia-Pacific. The United States led with 129 trials (58.1%), followed by China (*n* = 55, 24.8%), France (*n* = 6, 2.7%), Canada (*n* = 4, 1.8%), and Colombia, Australia, and Germany (*n* = 3 each). The remaining trials were distributed across South Korea (*n* = 2), and one trial each in Japan, Honduras, the United Kingdom, the Netherlands, Russia, South Africa, Spain, India, and Poland, totaling 17 countries represented in the dataset. Eight trials did not report a clearly identifiable primary country. Sponsorship was predominantly industry-driven: 166 trials (74.8%) were funded by industry, 53 (23.9%) by academic or other non-profit institutions, and 3 by government agencies including the National Institutes of Health (NIH). Among the most active sponsors, Beacon Therapeutics (*n* = 10), the Institute of Hematology and Blood Diseases Hospital, China (*n* = 9), Spark Therapeutics (*n* = 8), Ultragenyx Pharmaceutical (*n* = 7), Pfizer and 4D Molecular Therapeutics (*n* = 6 each) represented the highest trial volumes. The geographic concentration of trials in the United States and China, coupled with the predominantly industry-driven sponsorship structure, highlights a potential asymmetry in the availability of immunogenicity data across global populations, a consideration directly relevant to the generalizability of NAb seroprevalence estimates when extrapolated to underrepresented regions such as Latin America, sub-Saharan Africa, and South Asia.

#### 3.2.6 Safety and immunogenicity outcomes from long-term follow-up and observational studies

The 27 observational and long-term follow-up records retained as a reference group provide complementary evidence on the durability of therapeutic response and the immunological consequences of AAV vector administration. Several of these studies reported sustained transgene expression and clinical benefit over extended follow-up periods. The long-term follow-up of subjects treated with voretigene neparvovec-rzyl (Luxturna) for LCA due to RPE65 mutations (NCT02946879) documented stable visual function at five years post-treatment, supporting the durability of subretinal AAV2-mediated gene transfer in the immune-privileged ocular compartment. Similarly, long-term follow-up of fidanacogene elaparvovec recipients in hemophilia B (NCT03307980) demonstrated sustained factor IX expression over multi-year observation periods, with manageable immune-related hepatic transaminase elevations. Meanwhile, follow-up of DTX301 recipients with OTC deficiency (NCT03636438) remains ongoing, with outcomes focused on long-term safety and metabolic correction. Long-term follow-up of ADVM-022 for neovascular AMD (NCT04645212) and diabetic macular edema (NCT05607810) provides evidence on the durability of anti-VEGF transgene expression following intravitreal delivery, an approach designed to reduce the treatment burden associated with repeated anti-VEGF administrations. Notably, the AAVrh10-based gene therapy trial in hemophilia B (NCT02971969) demonstrated that immune-related elevations in hepatic transaminases were consistent with capsid-specific cellular immune responses rather than anti-transgene product immunity, highlighting the role of host immune responses against the AAV capsid as a key factor influencing transgene expression and durability.

Among the terminated or discontinued observational records, NCT03278873, a long-term follow-up study of AAV-based gene therapy for achromatopsia (CNGB3 and CNGA3) was terminated before completion of the planned follow-up period, while NCT01301573, a long-term follow-up study of rAAV-GAD treated subjects with Parkinson's disease, was also terminated; in both cases, no safety-related reason for termination was explicitly reported in the registry. Of particular relevance to the primary objective of this scoping review, NCT06311708 is an ongoing non-interventional study evaluating the seroprevalence of pre-existing antibodies against AAV9 in patients with PKP2-associated arrhythmogenic right ventricular cardiomyopathy (ARVC), providing disease-specific population-level data on neutralizing antibodies. Additionally, NCT05101902 was designed as a pre-screening eligibility study for a congenital adrenal hyperplasia gene therapy program, explicitly incorporating anti-AAV neutralizing antibody titers as a primary screening criterion, further supporting the role of pre-existing anti-AAV immunity as an operational constraint in clinical trial design.

## 4 Discussion, conclusions, and future directions

This scoping review integrates seroepidemiological evidence with an analysis of the global clinical trials registry to characterize pre-existing anti-AAV immunity as a determinant of feasibility, safety, and scalability in AAV-mediated gene therapy. A consistent pattern emerges across both datasets: the clinical pipeline is expanding, yet remains constrained by immunogenicity, small cohort sizes, and reliance on serotypes with high background seroprevalence. AAV2 remains the most frequently used defined serotype in clinical trials, consistent with its role as the canonical reference vector, although the largest proportion of trials now employs engineered or proprietary capsids, indicating a shift in vector design. The continued clinical use of AAV2 despite its relatively high background seropositivity may be attributed, at least in part, to factors beyond immune evasion, including its extensive clinical and preclinical characterization, established safety and regulatory experience (10), and comparatively mature manufacturing processes. These features may reduce translational and developmental uncertainty relative to newer, less extensively validated capsids, although pre-existing immunity remains an important limitation of AAV2-based gene therapy (3, 13). The serotypes most commonly used clinically (AAV2, AAV9, AAV5, AAV8) are also among the most extensively characterized and most frequently seropositive in human populations, indicating a misalignment between serotype selection and the underlying immune landscape. This pattern suggests that vector choice has been shaped largely by historical precedence and platform maturity rather than immune evasion, with NAbs acting as a structural bottleneck for patient eligibility, particularly given that seroprevalence against common serotypes such as AAV2 ranges from approximately 30% to 70% across adult populations globally (18, 21). The increasing adoption of engineered capsids reflects a shift toward more personalized gene therapy strategies, in which optimized serotype selection and tailored administration routes are considered alongside capsid engineering (36). The link between seroprevalence, trial feasibility, and clinical outcomes reflects an association rather than a demonstrated causal relationship, since the seroprevalence and registry datasets analyzed here are not directly linked at the individual patient level.

The clinical trial landscape further reflects these immunological constraints. The pipeline remains concentrated in early-phase development, with most trials in Phase 1 or Phase 1/2 designs and only a small proportion reaching Phase 3, reflecting both the novelty of the field and cumulative barriers to late-stage progression, including immunogenicity-related safety signals and challenges in sustaining transgene expression. That fewer than half of Phase 3 trials have posted results further indicates the limited availability of efficacy and immunogenicity data at advanced stages. Therapeutically, the predominance of ophthalmology among trial indications reflects the advantage of immune-privileged compartments, where local delivery (e.g., subretinal or intravitreal administration) reduces systemic immune exposure. Systemic indications, including hematological, hepatic, and neuromuscular diseases, are by contrast more susceptible to humoral and cellular immune responses (37).

This is particularly evident in liver-directed programs, where hepatotoxicity remains among the most clinically significant adverse events (37). Available evidence is consistent with a mechanism involving capsid-specific CD8+ T-cell responses against transduced hepatocytes, resulting in transaminase elevation and, in some cases, reduced transgene expression (46). Long-term observational records from this dataset (NCT02971969, NCT03307980) are consistent with capsid-directed cellular immunity as a contributor to declining efficacy; however, because these observations derive from registry reports rather than prospective mechanistic studies, this remains an association rather than an established mechanism.

The impact of pre-existing immunity is amplified by the small size of trial populations: median enrollment across interventional studies was 14 participants, with most trials targeting rare monogenic diseases. Under these conditions, standard NAb exclusion criteria can substantially reduce an already limited recruitment pool, as reflected in trial designs such as NCT05101902, which incorporates antibody screening as a primary eligibility step, and NCT06311708, a seroprevalence-focused protocol. This interpretation is further complicated by methodological heterogeneity in antibody detection: variability in assay design, sensitivity, and cutoff thresholds generates inconsistent prevalence estimates across studies (20, 47). The distinction between TAb and NAb assays carries clinical relevance, since NAbs are typically used for eligibility screening while TAbs have been hypothesized to reflect broader immune activation through mechanisms such as opsonization and complement engagement, which remain speculative based on the data synthesized here. Evidence from screening studies such as ECLIPSE (NCT04272554) has highlighted the potential limitations of relying solely on NAb testing, raising the possibility that TAb-positive, NAb-negative individuals may still be at risk of clinically relevant immune responses. However, whether this antibody profile is associated with reduced treatment efficacy remains to be prospectively established, supporting the need for integrated immunoprofiling (11, 48).

Geographic disparities further complicate interpretation and generalizability. Reported seroprevalence in regions such as sub-Saharan Africa and Latin America derives from a limited number of studies with heterogeneous methodologies (12, 21, 25) and is insufficient to support robust regional conclusions; these regions also remain underrepresented in both the literature and the clinical trial assays, in contrast to the United States and China, which together account for the majority of registered trials. This asymmetry introduces a translational gap, as seroprevalence estimates from high-income populations may not reflect immune landscapes in regions where future therapy deployment is expected. Twelve interventional trials in this dataset were terminated across indications including hemophilia, Parkinson's disease, Duchenne muscular dystrophy, and lysosomal storage disorders; while not all terminations are attributable to immunogenicity, immune-mediated hepatotoxicity, insufficient efficacy, and manufacturing limitations frequently co-occur (13).

Several complementary strategies are being pursued to address these challenges. Engineered capsids designed to evade pre-existing antibodies represent a major innovation axis (36). A 2025 review further catalogs capsid modification, empty-capsid decoys, and pharmacological adjuncts as complementary strategies to overcome pre-existing immunity (49). Capsid engineering has also been leveraged for goals beyond antibody evasion, including improved genome-editing precision through capsid mutants optimized for homology-directed repair delivery (50). Pharmacological co-administration strategies, such as pre-incubation with the peptide melittin, have similarly been explored to enhance rAAV2 transduction efficiency, although direct insertion of the peptide into the capsid itself reduced rather than improved performance (51); while these modifications were not designed primarily to reduce antibody binding, they illustrate the breadth of ongoing capsid innovation relevant to next-generation vector design. Immunomodulatory approaches, including enzymatic IgG degradation with imlifidase (NCT06518005), plasmapheresis, and transient immunosuppression, aim to reduce antibody burden and enable vector administration (2, 47). Emerging technologies such as dual IgM/IgG-cleaving enzymes (e.g., IceMG) extend this approach by targeting additional layers of the humoral response, including complement activation and B-cell receptor signaling (52). The long-term safety, durability, and scalability of these strategies remain to be established in larger clinical cohorts.

Beyond antibody prevalence, the immune mechanisms engaged by AAV vectors differ across serotypes and operate on distinct timescales, with important implications for capsid engineering and immunomodulatory strategies. AAV2, one of the most extensively characterized serotypes in this review and one to which humans are commonly exposed, is subject to substantial pre-existing humoral immunity, with IgG representing the predominant antibody response against the capsid (11, 17). Independently of pre-existing antibody levels, AAV vector genomes can also activate innate pattern-recognition pathways, most notably Toll-like receptor 9 (TLR9), through recognition of unmethylated CpG motifs, thereby promoting downstream inflammatory and adaptive immune responses following administration (11). At the cellular level, capsid-derived peptides can be presented on MHC class I molecules and trigger capsid-specific CD8+ T-cell responses, which have been associated with hepatotoxicity and loss of transgene expression in some liver-directed AAV programs (46, 48). These immune mechanisms are not mutually exclusive: pre-existing NAbs can interfere with vector entry, innate sensing can occur shortly after administration, and cellular immune responses may emerge over subsequent days to weeks (11, 17). This distinction has practical implications for vector design and immune management. Strategies aimed at reducing NAb binding, including capsid epitope modification and bioengineered variants, primarily address the humoral barrier, whereas vector-genome modifications such as CpG depletion can reduce innate immune activation, and transient immunosuppression may help limit downstream cellular immune responses (17, 49). Thus, understanding which immune mechanisms predominate for a given serotype, route of administration, and clinical context, rather than considering antibody seroprevalence in isolation, is critical for guiding rational capsid design and the selection of complementary immunomodulatory strategies.

### 4.1 Strengths, limitations, and future directions

A principal strength of this review lies in its dual analytical framework, integrating seroprevalence data with a comprehensive and up-to-date analysis of the global AAV clinical trial pipeline. This approach allows immunological considerations to be examined alongside their potential implications for clinical trial design, recruitment, and outcomes. However, the analysis is limited by methodological heterogeneity across studies, lack of assay standardization, and geographic bias in both published and registry data. Reliance on registry-reported information may also introduce inconsistencies due to incomplete or non-standardized reporting.

A further limitation is that the clinical trial extraction framework (Section 2.2.2) did not systematically capture route of administration, vector dose, or immunosuppression protocols as structured variables. Consequently, these factors could not be evaluated alongside seroprevalence and clinical outcomes within the present dataset. This limitation is relevant because previous studies have identified vector design, dose, route of administration, and immunosuppression as important determinants of AAV immunogenicity, acting alongside pre-existing antibody status to shape the overall immune response (53). Systemically administered, particularly high-dose regimens, have generally been associated with more pronounced innate and adaptive immune responses than locally restricted or relatively immune-privileged routes, such as intraocular or subretinal delivery, in previous studies. Immunosuppression protocols also vary across clinical trials, with no standardized approach linking regimen selection to vector serotype or dose. Because these variables were not part of our original extraction framework, we could not retrospectively stratify the 222-trial dataset without re-abstracting each record. We therefore identify their systematic assessment as a priority for a dedicated follow-up analysis rather than addressing these factors within the scope of the present scoping review.

As an exploratory assessment of the completeness of publicly available trial information, we screened the intervention descriptions of the 222 interventional trials for explicit reporting of administration route and immunosuppressive co-interventions. Only 11 trials (5.0%) explicitly reported the vector administration route: 6 described systemic intravenous delivery, primarily in hematologic and metabolic or lysosomal indications, whereas 5 described locoregional delivery, including intrathecal or stereotactic CNS infusion for neurological targets and subretinal or intravitreal injection for ophthalmologic targets. Separately, 11 trials (5.0%) reported a corticosteroid or other immunosuppressive agent as a co-intervention, most commonly prednisone/prednisolone or corticosteroid taper regimens. One hepatic-directed trial (NCT06518005) reported the use of imlifidase, an IgG-cleaving enzyme, as a pre-treatment strategy to enable vector administration in seropositive patients. These findings are presented descriptively and should not be interpreted as a formal analysis of the relationship between administration route, immunosuppression, and clinical outcomes. Rather, they highlight the limited availability and heterogeneity of these data in publicly accessible trial records. Among the trials that reported administration route, systemic delivery was primarily described for hematologic and metabolic or lysosomal targets, whereas locoregional approaches were reported for CNS and ophthalmologic targets, broadly reflecting established tissue-specific delivery practices. Immunosuppressive co-interventions were reported across several therapeutic areas, including hepatic, metabolic, neurological, cardiovascular, and neuromuscular indications, without a clear concentration in a single therapeutic area given the small number of reported cases. Importantly, the absence of information in the registry should not be interpreted as evidence that these interventions were not used. Instead, the limited reporting underscores the need for more systematic capture of administration route, vector dose, and immunosuppression in future AAV clinical trial datasets

Future research should prioritize four key areas: (i) development of standardized, regulatory-grade assays for TAb and NAb quantification (54); (ii) expansion of population-representative seroprevalence studies in underrepresented regions; (iii) integration of prospective immune monitoring into late-phase clinical trials to establish robust correlates of efficacy and toxicity; and (iv) comparative evaluation of mechanism-based immunomodulatory strategies, including IgG depletion for pre-existing humoral immunity, genome engineering to reduce innate immune activation, and transient immunosuppression to limit cellular immune responses.

Pre-existing immunity to AAV vectors constitutes a fundamental barrier to gene therapy, influencing multiple stages of clinical development, from patient screening to long-term efficacy. Although the clinical pipeline continues to expand, its broader application remains constrained by immunogenicity, limited cohort sizes, and geographic imbalances in data generation. Addressing these challenges will require coordinated advances in assay standardization, capsid engineering, and clinical trial design, with greater integration of immunological insights into translational strategies to achieve safe, effective, and globally accessible AAV-based therapies.
