# Supplementary material for: Global seroprevalence of neutralizing and total antibodies against AAV vectors and their impact on the clinical gene therapy landscape: a scoping review
Source: Front Med (Lausanne). 2026 Sep 22;13:1925844. doi: 10.3389/fmed.2026.1925844 (PMC13639339; doi:10.3389/fmed.2026.1925844)
Supplement: Supplementary file 1 [file Table1.docx]

Supplementary Material

Global Seroprevalence of Neutralizing and Total Antibodies Against AAV Vectors and Their Impact on the Clinical Gene Therapy Landscape: A Scoping Review

Cristina Sanchez-Gutierrez, Luisa F. Bohorquez-Zambrano, M. Camila Garnica, Melvin Y Rincon, Augusto Elías Valderrama-Aguirre

## 1.1 Supplementary Information 1. Summary of research articles included in the scoping review

Summary of the 50 research articles included in the scoping review that report the global prevalence of TAbs and NAbs against AAV vectors across various populations. NE: Not evaluated; NA: Not available; OD: Optical density; yr: Years; m: Months.

| **Year** | **Method** | **Country** | **Health**  **condition** | **N** | **Age** | **Serotypes** | **Higher**  **TAbs**  **prevalence** | **Lower**  **TAbs**  **prevalence** | **Higher NAbs**  **prevalence** | **Lower NAbs**  **prevalence** | **Higher prevalence by groups** | **Lower prevalence by groups** | **References** |
| --- | --- | --- | --- | --- | --- | --- | --- | --- | --- | --- | --- | --- | --- |
| 1968 | NAbs | USA | Healthy donors | 52 | NA | AAV1, AAV2, AAV3, AAV4 | NA | NA | AAV2 ≈ 40%; titer <0.32 | AAV4= 0% ; titer <0.32 | Young adults AAV2 ≈ 50% | Children <1-2 yr; AAV4=0% | (1) |
| 1999 | TAbs/  NAbs | USA | Cystic fibrosis (CF) (n=29); Healthy donors (n=45) | 74 | 18-54yr | AAV2 | AAV2 (IgG) = 96% ; 1:200 | NA | AAV2=32%; titer 1:20 | NA | NA | NA | (2) |
| 1999 | TAbs/ NAbs | Brazil, Germany, Japan, Switzerland | Healthy donors | 787 | 16-59 yr | TAbs: AAV2; NAbs: AAV2, AAV3, AAV5 | AAV2 (IgG) = 73% (50-59 yr) | AAV2 (IgG) = 49% (20-29 yr) | AAV2 = 84.37%; titer NA | AAV5= 37.5%; titer NA | Brazil AAV2 (IgG) = 56% | Japan AAV2 (IgG) = 46% | (3) |
| 2006 | NAbs | USA | Cystic fibrosis  (n=129) and Non CF subjects (n=37) | 166 | ≈ 19.5 yr | AAV2, AAV5, AAV6 | NE | NE | AAV2 (adults) ≈ 30%; titer 1:20 | AAV5  <5%; titer 1:20 | AAV2 , AAV6 (Non CF ≥ 18)  ≈ 30%; titer 1:20 | AAV5 (CF, 0-10 yr) <5%; titer 1:20 | (4) |
| 2009 | NAbs | Australia, Belgium, Greece, Italy, Kenya, Rwanda, South Africa, Uganda, USA, Zambia | Healthy donors | 888 | NA | AAV1, AAV2, AAV7, AAV8, AAVrh32.33 | NE | NE | AAV2 ≈60%; titer >1:20 | AAVrh32.33 = <2%; titer >1:20 | Uganda AAV2 ≈ 63%; titer: 1:20 | Belgium, Kenya, Greece, Italy AAVrh32.33 = 0%, titer 1:80 | (5) |
| 2010 | TAbs/ NAbs | France | Healthy donors | 226 | 25-64 yr | AAV1, AAV2, AAV5, AAV6, AAV8, AAV9 | AAV2 (IgG) ≈ 72%;  titer ≥1:30 | AAV8 (IgG) ≈ 38%; titer ≥1:30 | AAV2 = 59%; titer 1:20 | AAV5 = 3.2%; titer 1:20 | NA | NA | (6) |
| 2011 | NAbs | USA | Healthy donors (0-18 yr) | 752 | 0-18 yr | AAV2, AAV8 | NE | NE | AAV2 =22.1%;  titer ≥1:20 | AAV8= 15.7%; titer ≥1:20 | Group 3-18 yr AAV2= 21.5%; titer ≥1:40 | Group <1 yr AAV5 ≈15%; titer ≥1:40 | (7) |
| 2011 | NAbs | Netherlands | Healthy donors (n=100), inflammatory bowel disease (n=200) | 300 | 16-84 yr | AAV1, AAV2, AAV3, AAV4, AAV5, AAV6, AAV8 | NE | NE | AAV8 (HD) = 94%; AAV6 (IBD) = 85%; titer NA | AAV4(HD)= 31%; AAV4 (IBD) = 44%, titer NA | Patients with extraintestinal manifestations | AAV1, AAV5, AAV8 IBD patients | (8) |
| 2012 | NAbs; 4-year follow-up | USA | Hemophilia A pediatric  (n=62) | 277  ( ≈ 4.5 per patient) | 9-40 months | AAV2, AAV5, AAV8 | NE | NE | AAV2= 43.5%;  titer 1:20 | AAV8= 22.6%; titer 1:20 | Group ≥5 yr (AAV2)= 27% | Group <2 yr (AAV8) ≈ 3% | (9) |
| 2012 | TAbs/ NAbs | France | Healthy donors | 55 | NA | AAV1 | AAV1 (IgG)= 64.7%; titer 1:3 to 1:65610 | NE | AAV1= 58.8%; titer 1:2 to 1:3200 | NE | NA | NA | (10) |
| 2013 | NAbs | China | Healthy donors | 500 | <18-56 yr | AAV1 | NE | NE | AAV1=69.8%; titer 1:10 | NE | Gender female (AAV1)= 74.8%;  Anhui city= 82%; titer 1:10 | Gender male (AAV1)= 65.8%; titer 1:10 | (11) |
| 2014 | NAbs | Austria, Canada, China, England, France, Germany, Italy, Russia, Spain, Ukraine and USA | Healthy donors  (n=500) and HIV-1-infected (n= 270) | 770 | 18-74 yr | AAV2, AAV5, AAV8 | NE | NE | AAV2 96.6%; titer 1:10 | AAV5 40.2%; titer 1:10 | Group <18 yr AAV2 = 100%; Healthy AAV5= 40.6%; titer 1:10 | Group 31-40 yr AAV5 = 37%; HIV-1 infected AAV5 =37%; titer 1:10 | (12) |
| 2014 | NAbs in vitro human and pigs | NA | Human individuals (n=31) and Yorkshire pigs (n=66) | 97 | NA | BNP116 AAV | NE | NE | Humans BNP116= 68%; titer >1:8 | Pigs BNP116= 56%; titer >1:8 | NE | NE | (13) |
| 2014 | NAbs | Japan | Healthy donors (HD= 85) and hemophilia  (n=59) | 144 | 12 to ≥ 52 yr | AAV1, AAV2, AAV5, AAV8, AAV9 | NE | NE | AAV1= 39.7%; titer ≥1:224 | AAV9=27.4%; titer ≥1:224 | Hemophiliacs group >52  yr: AAV1, AAV2, AAV8= 100%, titer ≥1:224 | HD group 12-21 yr: AAV1, AAV2, AAV8 <5%; titer ≥1:224 | (14) |
| 2015 | TAbs/NAbs | Spain | Healthy donors (HD) | 100 | NA | AAV2, AAV9, AAVrh.10 | AAV2 (IgG)= 72%; AAVrh.10 (IgG)= 59%; OD= 1000 >1600 | AAV9 (IgG)= 47%; OD= ≤1000 >1600 | AAV2= 71%; AAVrh.10= 21%; titer 1:20 | AAV9= 18%; titer 1:20 | NA | NA | (15) |
| 2015 | NAbs | China and USA | Healthy donors from China (n=100) and adults American as controls (n=30) | 130 | ≤ 35 to 52 yr | AAV2, AAV3, AAV8, AAVLK03 | NE | NE | AAV2= 92%; AAVLK03= 91%; titer ≥ 1:20 | AAV3= 89%; AAV8= 69%; titer ≥ 1:20 | American adults AAV3= 66.7%; titer ≥ 1:80; Gentleness type n= 33% | Yang-deficiency type constitution= 29% | (16) |
| 2015 | NAbs | Italy, Netherlands, Turkey | Mucopolysaccharidosis VI (MPS VI) | 36 | 5-25 yr | AAV8 | NE | NE | AAV8 42%; titer ≥1:5 | NE | Group 19-25 yr= 67%; Asiatic origin = 50%; titer ≥1:5 | Group 5-10 yr=  31%; European and African origins = 33%; Italian center= 14%; titer ≥1:5 | (17) |
| 2016 | NAbs *in vitro* and *in vivo* | Belgium, Denmark, Germany, Hungary, Israel, Netherlands, Poland, Sweden, UK, USA | Healthy donors (HD=30) and chronic systolic heart failure (HF=30 in pilot study; HF= 1552 CUPID-2 trial ) | 1612 | 60.5 ± 11.5 yr | AAV1, AA2 | NE | NE | Pilot study: AAV1=  66.7 %; ≥1:4. CUPID-2 trial AAV1= 59.5%; titer  ≥1:2. | Pilot study: AAV2= 40% (HF); titer ≥1:4 | Hungary and Poland: AAV1= 79%; Europe AAV1= 67.7%; Group ≥ 71 yr AAV1= 66%; titer ≥1:2. | Sweden: AAV1= 48% ; USA AAV1= 53.5%;  Group 31-40 yr AAV1= 41%; titer ≥1:2 | (18) |
| 2017 | NAbs | USA | Healthy donors (HD= 38) and primary Sjögren’s (pSS= 39) | 77 | 17-75 yr; | AAV2, AAV4, AAV5, AAV12, BAAV (bovine AAV) | NE | NE | pSS AAV2 and BAAV  =62%; titer 1:25 | HD AAV12= 0%; pSS AAV12= 8%; titer 1:25 | pSS AAV2 ≈60%; titer >1:201 | pSS HD= AAV5, AAV12, BAAV =0%; titer >1:201 | (19) |
| 2017 | TAbs/ NAbs in vitro human | USA | Healthy donors (HD) and hemophilia A (HA) | 124 | HD 20-63 yr; HA 21-68 yr | AAV5 | AAV5 17% | NE | AAV5=17%; titer 1:01 | NE | HD: AAV5 TABS+ NAbs+ =18% | HA: AAV5 TAbs+ NAbs+ =13% | (20) |
| 2019 | TAbs/ NAbs | France, Germany, Italy and Netherlands | Crigler-Najjar (CN) | 49 | 5-26 yr, mean 20 yr | AAV2, AAV5, AAV8 | Anti-AAV8 NAb titer vs total IgG= R² 88% | Anti-AAV2 NAb titer vs total IgG= R² 56% | AAV8 30.6%; titer 1:1 - 1:1000 | No prevalence 69.4% | NE | NE | (21) |
| 2019 | TAbs/ NAbs | France (Other countries not mentioned) | Healthy donors (HD=113) and Duchenne Muscular Dystrophy (DMD=130) | 243 | 3 to >12 yr | AAV1, AAV2, AAV8 | AAV2 52% and 55% of IgG positive HD and DMD subjects; titer ≥ 1:3 | AAV8 39% and 44% of IgG positive HD and DMD subjects; titer ≥1:3 | AAV2 64% and 56% in HD and DMD patients; titer >1:200 | AAV8 6% and 15% in HD and DMD patients; titer >1:200 | DMD patients AAV2 = 44%; titer 1:200 | HD AAV8 =64%; titer 1:200 | (22) |
| 2019 | NAbs; 4 year follow-up | Australia | Nondiabetic patients (n=45) and type 1 diabetes (T1DM) (n=72) | 117 | 18-77 yr | AAV2, AAV5, AAV6, AAV8 | NE | NE | AAV2= 61-81%; titer 1:100 | AAV5 and AAV8= 2-10%; AAV6= 27-31%; titer 1:1000 | Diabetic group, AAV2≈ 2-10%; titer 1:10000 | AAV5 and AAV6= 0%; titer 1:10000 | (23) |
| 2019 | TAb/ NAbs | UK | Hemophilia A (HA) | 100 | 27‐57 yr | AAV5, AAV8 | AAV8= 23%; titer >1:15 | AAV5= 21%; >titer 1:15 | AAV8 38%; titer 3.35 to >256 | AAV5 25%; titer <2 to >256 | With Hepatitis C status: AAV8 NAbs= 50%; TAbs+ NAbs+  AAV5= 16% | TAb+ NAbs+  AAV8= 21% | (24) |
| 2019 | TAb/  NAbs | UK | Anonymized | 323 | <6 months to 84 yr | TAbs: AAV-LK03; NAbs: AAV3B, AAV8 | AAV-LK03 (IgG)= 39%; titer 1:2 | Group 7m-2yr: AAV-LK03= 28%; titer 1:2 | AAV3B= 35%; AAV-LK03= 23%; titer 1:5 | AAV8=18%; titer 1:5 | Gender male: AAV3B ≈40%; Group >18 yr AAV3B ≈50%; titer >1:5 | Gender male: AAV-LK03≈ 15%; Group 3-17 yr AAV8 <5%; titer >1:5 | (25) |
| 2019 | NAbs | Austria, Germany | Healthy donors | 60 | NA | AAV8 | NE | NE | AAV8= 43%; titer ≥1:80 | NE | NA | NA | (26) |
| 2019 | TAbs/ NAbs | Europe Cohort (Austria),  USA | Healthy donors (HD) and Hemophilia B (HB) | 209 | 19-84 | TAbs: AAV8; NAbs: AAV1, AAV2, AAV5, AAV8 | AAV8 (HD)= 38%, (HB)= 41%; titer ≥1:5 | USA 1 (HD), AAV8≈ 25%; titer ≥1:5 | AAV8 (HD)= 83%; AAV2 (HD and HB); 47%–74%; titer 1:5 | AAV5= 20%–59%; titer 1:5 | EU3 group (HD) AAV2 ≈ 70%; titer ≥1:5 | HD and HB groups AAV1= 0%; titer 1:5 | (27) |
| 2019 | NAbs | South Korea | Vitreoretinal diseases | 32 | 29-75 yr; | AAV2, AAV5, AAV8, AAV9 | NE | NE | AAV2 (Sera) ≈ 98%; AAV2 (Vitreous) ≈ 52%; titer ≥1:20 | AAV8 (Sera) ≈ 37%; AAV8 (Vitreous) = 0%; titer ≥1:20 | NAbs in sera | NAbs in the vitreous humour | (28) |
| 2019 | NAbs | USA | Healthy donors (HD) and Hemophilia | 46 | NA | AAV2, AAV3B, AAV7, AAV8, AAV9 | NE | NE | AAV2= 69.6%; AAV7= 67.4%; AAV3B= 52.2%; titer ≥1:20 | AAV8= 37%; AAV9= 23.9%; titer ≥1:20 | AAV2 and AAV3B (HD)= 60%; AAV2 (H)= 76.9% | AAV9 (HD, H)= 25%, 23.1% | (29) |
| 2021 | NAbs | Australia | Post-mortem eye cups | 80 | 20-88 yr; | AAV2, AAV5, AAV6, AAV8 | NE | NE | AAV6= 66%; AAV2= 52.5%; titer 1:2 | AAV8 2.5%, AAV5= 23%; titer 1:2 | No differences between gender | NA | (30) |
| 2021 | TAbs/NAbs | India | Healthy donors (HD) and Hemophilia B (HB) | 235 | Healthy donors=  3 to ≥18yr, Hemophilia B ≥18 yr | AAV3 | AAV3 (HB), 92.4%; OD= 0.7 | AAV3 (HD), 79.7%; OD= 0.7 | AAV3 (HB), 91.3%; titer >1:10 | AAV3 (HD), 65%; titer >1:10 | HB: ≥36 yr, 93.5%; titer >1:5 | HD: 2-5 yr, 34.6%; titer >1:5 | (31) |
| 2022 | TAbs/NAbs | Germany | Donors with suspected previous AAV2 infections | 24 | 11-61 yr; mean 41 | AAV2WT, AAV2.GL, AAV2.NN | AAV2= 91.7%; titer 1:400 | AAV2.NN = NA; titer 1:400 | AAV2WT= 65.2%; titer >1:40 | AAV2.GL= NA; titer >1:40 | AAV2WT= 65.2%; titer >1:40 | Slightly younger (mean 35.6 yr) AAV2W | (32) |
| 2022 | TAbs/ NAbs | Germany | Healthy donors | 40 | 21-63 yr | AAV2, AAV8, AAV9 | AAV2 (IgG)= 37.5%; titer 1:5 | AAV8 (IgG) and AAV9 (IgG) | AAV2 45%; titer 1:4 | NA | NE | NE | (33) |
| 2022 | NAbs | Japan | Healthy donors (HD=100), hemophilia A (n=175) and B (n=41) | 316 | mean 42-43 yr | AAV1, AAV2, AAV3B, AAV5, AAV6, AAV7, AAV8, AAV9, AAVrh10 | NE | NE | HD AAV2 = 27%; Hemophiliacs AAV3B =29.2%; titer 1:1 | HD AAV9 =20%;  Hemophiliacs AAV8= 20.4%; titer 1:1 | 60s or older age group (HD) AAV2 ≈ 65%; Hemophiliacs AAV3B ≈ 70% patients | Younger HD (20's yr) AAV6 = 0%; Hemophiliacs (40's yr) AAV9 <5% | (34) |
| 2022 | NAbs | UK | Hemophilia B | 19-69 yr; mean 47yr | 43 | AAV6 | NE | NE | AAV6=30.6%; titer 1:10 | NE | Hepatitis C exposure AA6= 60% | NE | (35) |
| 2022 | NAbs in vitro human | USA | Healthy donors (HD=19) and methylmalonic acidemia (MMA=48) | 37 | Mean age 47yr (HD); 3-38 yr (MMA) | AAV2, AAV9, AAV44.9 | NE | NE | AAV2= 37%;  titer ≥ 1:16 | AAV44.9= 27%; titer ≥ 1:16 | HD AAV2= 37% | MMA patients AAV44.9= 13% | (36) |
| 2022 | TAb/ NAbs | India | Hemophilia A (HA) | 521 | 0 to ≥18 yr | AAV3 | AAV3=96%; 0.7 OD | NE | AAV3= 77.5%; titer ≥ 1:10 | NE | AAV3(≥ 36 yr)= 90.8%; titer ≥ 1:10 | AAV3 (2-5 yr) =39.1%; titer ≥ 1:10 | (37) |
| 2022 | NAbs | China | Hemophilia B | 10 | NA | AAV5, AAV8, AAV9, AAVzk2 | NE | NE | AAV8 and AAV9 = 40%; titer 1:4 | AAV5 and AAVzk2= 0%; titer 1:4 | NE | NE | (38) |
| 2023 | NAbs | Colombia | Healthy donors (HD) and heart failure (HF) patients | 120 | 51-72 yr | AAV1, AAV2, AAV9 | NE | NE | AAV2 (HF)=43%; AAV2 (HD)= 45%; titer ≥1:50 | AAV9 (HF) = 20%; AAV9 (HD) =23.33%; titer ≥1:50 | AAV2 (HD) 45%; titer ≥1:50 | AAV9  (HF) 20%; titer ≥1:50 | (39) |
| 2023 | NAbs | USA | Healthy donors | 50 | NA | AAAV (Avian AAV), AAV2, AAV5, AAV9 | NE | NE | AAV2 46%; titer: 1:1 | AAV5 12%; titer: 1:1 | NE | NE | (40) |
| 2023 | NAbs | USA | Osteoarthritis (OA) | 54 | 44-79 yr | AAV2, AAV2.5; AAV5 | NE | NE | AAV2=57.14%; titer >1:1000 | AAV2.5=16%; titer >1:1000 | NA | Mid-stage and advanced OA AAV2.5= 13%; titer >1: 1000 | (41) |
| 2023 | TAbs/ NAbs | USA | Duchenne muscular dystrophy (DMD) | 101 | 2–20 yr; mean 11 yr | AAV2, AAV8, AAV9, AAVrh74 | No OD450 differences AAV2, AAV8, and AAVrh74 | AAV8= NA; titer 1:240 | AAV2= 56%; AAV8= 47%; titer >1:240 | AAV9= 36%; AAVrh74= 32%; titer 1:5 to 1:20 | African American AAV8= 67% | Caucasian AAVrh74= 19% | (42) |
| 2024 | NAbs | Australia, Canada, France, Germany, Italy, Japan, South Korea, Spain, UK and USA | Cancer, DMD,  and other diseases not listed in the provided contexts | 552 | 48.6 yr | AAV1, AAV5, AAV6, AAV8, AAV9, AAVRh74var | NE | NE | AAV1 adults 74.9%; titer 1:1 | AAV9 adults 57.8%; titer 1:1 | South Korea AAV1=96%; titer 1:1 | Japan AAVRh74var 36%; titer 1:1 | (43) |
| 2024 | TAbs/ NAbs ≤3 years follow-up | Austria, France, Germany, Italy, Spain and USA | HA (n=194) and B (n=98) | 242 (Baseline) | 18-75 yr; mean 35.3 yr | AAV2, AAV5, AAV8 | AAV8 at baseline 42.3%; titer 1:20 | AAV5 at baseline 38.1%; titer 1:20 | AAV5 53.4%; titer 1:5 | AAV8 46.9%; titer 1:5 | HB at baseline AAV2 NAbs 59.6%; Spain AAV5= 65% | HB AAV8 45.8%. Italy AA8 and AAV5 =33.3% | (44) |
| 2024 | NAbs | China | DMD | 100 | 3-15 yr; mean 8 yr | AAV9 | NE | NE | AAV9 42%; titer 1:4 | Group <4 yr AAV9= 0%; titer 1:4 | Shandong and Henan >60% | Beijing and Tianjin <30% | (45) |
| 2024 | NAbs | China | HD, DMD, Becker muscular dystrophy (BMD), adults controls (AC), schoolchild controls (SC) | 733 | mean age: 9 yr | AAV2, AAV9 | NE | NE | AAV2 85.8%; titer 1:1 | AAV9 55.9%; titer 1:1 | AC group AAV9 100%; SC group AAV2 97.4%; DMD/BMD AAV9 86.6% | SC group AAV9 17%; AC group AAV2 31.9%; DMD/BMD AAV2 66.7% | (46) |
| 2025 | NAbs | USA | Niemann-Pick disease, type C1 | 44  (2  per individual) | 0.9-17.4 years at time 1 and 5.8-29.4 years at time 2 | AAV2, AAV9 | NE | NE | AAV9 40.9% (time 1), 36.4% (time 2); titer ≥1:5 | AAV2 31.8% at both time points; titer ≥1:5 | NA | NA | (47) |
| 2025 | TAbs/ NAbs | China | HD (n=270) and patients with rare diseases (n=71) | 341 | mean age: 33.9 yr | AAV9 | AAV9 (IgG)= 64.8% healthy donors; titer ≥1:100 | AAV9 (IgG)= 62% patients with rare diseases; titer ≥1:100 | AAV9 58.7%; titer ≥1:100 | NE | Healthy donors 59.3% | Patients with rare diseases 56.3% | (48) |
| 2025 | TAbs/ NAbs | Belgium, Germany, Ireland, Italy, Netherlands, Sweden, UK, and USA | Male patients (≥18 yr) with hemophilia B | 67 | mean age: 43 yr | AAV5 | AAV5 (IgM)=9%; (IgG) ≈48%; titer ≥1:50 | NE | AAV5 48%; titer ≥1:2 | NE | NAbs  ≥51yr 75% | NAbs 31-40 yr 28.6% | (49) |
| 2026 | TAbs/ NAbs | Spain | Healthy donors | 165 | NA | TAbs: AAV2, AAV5, AAV6, AAV8, AAV9 NAbs: AAV6, AAV8, AAV9 | AAV6 (IgG)= 84.4%; AAV2 (IgG)= 74.4%; AAV8 (IgG)= 60% | AAV9 (IgG)= 51.2%; AAV5 (IgG)= 40.4% | AAV6= 62.8%; AAV8= 60.4% | AAV9= 52.4% | NA | NA | (50) |

**References**

1.	Blacklow NR, Hoggan MD, Rowe WP. Serologic Evidence for Human Infection With Adenovirus-Associated Viruses. JNCI: Journal of the National Cancer Institute. 1968 Feb 1;40(2):319–27. doi:10.1093/jnci/40.2.319

2.	Chirmule N, Propert KJ, Magosin SA, Qian Y, Qian R, Wilson JM. Immune responses to adenovirus and adeno-associated virus in humans. Gene Ther. 1999 Sep;6(9):1574–83. doi:10.1038/sj.gt.3300994

3.	Erles K, Sebökovà P, Schlehofer JR. Update on the prevalence of serum antibodies (IgG and IgM) to adeno-associated virus (AAV). J Med Virol. 1999 Nov;59(3):406–11. doi:10.1002/(sici)1096-9071(199911)59:3<406::aid-jmv22>3.0.co;2-n PubMed PMID: 10502275.

4.	Halbert CL, Miller AD, McNamara S, Emerson J, Gibson RL, Ramsey B, et al. Prevalence of neutralizing antibodies against adeno-associated virus (AAV) types 2, 5, and 6 in cystic fibrosis and normal populations: Implications for gene therapy using AAV vectors. Hum Gene Ther. 2006 Apr;17(4):440–7. doi:10.1089/hum.2006.17.440 PubMed PMID: 16610931; PubMed Central PMCID: PMC4292890.

5.	Calcedo R, Vandenberghe LH, Gao G, Lin J, Wilson JM. Worldwide Epidemiology of Neutralizing Antibodies to Adeno-Associated Viruses. J Infect Dis. 2009 Feb 1;199(3):381–90. doi:10.1086/595830 PubMed PMID: 19133809; PubMed Central PMCID: PMC10826927.

6.	Boutin S, Monteilhet V, Veron P, Leborgne C, Benveniste O, Montus MF, et al. Prevalence of Serum IgG and Neutralizing Factors Against Adeno-Associated Virus (AAV) Types 1, 2, 5, 6, 8, and 9 in the Healthy Population: Implications for Gene Therapy Using AAV Vectors. Human Gene Therapy. 2010 Jun;21(6):704–12. doi:10.1089/hum.2009.182

7.	Calcedo R, Morizono H, Wang L, McCarter R, He J, Jones D, et al. Adeno-associated virus antibody profiles in newborns, children, and adolescents. Clin Vaccine Immunol. 2011 Sep;18(9):1586–8. doi:10.1128/CVI.05107-11 PubMed PMID: 21775517; PubMed Central PMCID: PMC3165215.

8.	van der Marel S, Comijn EM, Verspaget HW, van Deventer S, van den Brink GR, Petry H, et al. Neutralizing antibodies against adeno-associated viruses in inflammatory bowel disease patients: implications for gene therapy. Inflamm Bowel Dis. 2011 Dec;17(12):2436–42. doi:10.1002/ibd.21673 PubMed PMID: 21370319.

9.	Li C, Narkbunnam N, Samulski RJ, Asokan A, Hu G, Jacobson LJ, et al. Neutralizing antibodies against adeno-associated virus examined prospectively in pediatric patients with hemophilia. Gene Ther. 2012 Mar;19(3):288–94. doi:10.1038/gt.2011.90 PubMed PMID: 21697954.

10.	Veron P, Leborgne C, Monteilhet V, Boutin S, Martin S, Moullier P, et al. Humoral and cellular capsid-specific immune responses to adeno-associated virus type 1 in randomized healthy donors. J Immunol. 2012 Jun 15;188(12):6418–24. doi:10.4049/jimmunol.1200620 PubMed PMID: 22593612.

11.	Liu Q, Huang W, Zhao C, Zhang L, Meng S, Gao D, et al. The prevalence of neutralizing antibodies against AAV serotype 1 in healthy subjects in China: Implications for gene therapy and vaccines using AAV1 vector. Journal of Medical Virology. 2013;85(9):1550–6. doi:10.1002/jmv.23647

12.	Liu Q, Huang W, Zhang H, Wang Y, Zhao J, Song A, et al. Neutralizing antibodies against AAV2, AAV5 and AAV8 in healthy and HIV-1-infected subjects in China: implications for gene therapy using AAV vectors. Gene Ther. 2014 Aug;21(8):732–8. doi:10.1038/gt.2014.47

13.	Ishikawa K, Fish KM, Tilemann L, Rapti K, Aguero J, Santos-Gallego CG, et al. Cardiac I-1c overexpression with reengineered AAV improves cardiac function in swine ischemic heart failure. Mol Ther. 2014 Dec;22(12):2038–45. doi:10.1038/mt.2014.127 PubMed PMID: 25023328; PubMed Central PMCID: PMC4429688.

14.	Mimuro J, Mizukami H, Shima M, Matsushita T, Taki M, Muto S, et al. The prevalence of neutralizing antibodies against adeno-associated virus capsids is reduced in young Japanese individuals. J Med Virol. 2014 Nov;86(11):1990–7. doi:10.1002/jmv.23818 PubMed PMID: 24136735.

15.	Thwaite R, Pagès G, Chillón M, Bosch A. AAVrh.10 immunogenicity in mice and humans. Relevance of antibody cross-reactivity in human gene therapy. Gene Ther. 2015 Feb;22(2):196–201. doi:10.1038/gt.2014.103

16.	Ling C, Wang Y, Feng Y lu, Zhang Y ni, Li J, Hu X rui, et al. Prevalence of neutralizing antibodies against liver-tropic adeno-associated virus serotype vectors in 100 healthy Chinese and its potential relation to body constitutions. J Integr Med. 2015 Sep;13(5):341–6. doi:10.1016/S2095-4964(15)60200-X PubMed PMID: 26343106.

17.	Ferla R, Claudiani P, Savarese M, Kozarsky K, Parini R, Scarpa M, et al. Prevalence of anti-adeno-associated virus serotype 8 neutralizing antibodies and arylsulfatase B cross-reactive immunologic material in mucopolysaccharidosis VI patient candidates for a gene therapy trial. Hum Gene Ther. 2015 Mar;26(3):145–52. doi:10.1089/hum.2014.109 PubMed PMID: 25654180; PubMed Central PMCID: PMC4367235.

18.	Greenberg B, Butler J, Felker GM, Ponikowski P, Voors AA, Pogoda JM, et al. Prevalence of AAV1 neutralizing antibodies and consequences for a clinical trial of gene transfer for advanced heart failure. Gene Ther. 2016 Mar;23(3):313–9. doi:10.1038/gt.2015.109

19.	Corden A, Handelman B, Yin H, Cotrim A, Alevizos I, Chiorini JA. Neutralizing antibodies against adeno-associated viruses in Sjögren’s patients: implications for gene therapy. Gene Ther. 2017 Apr;24(4):241–4. doi:10.1038/gt.2017.1

20.	Falese L, Sandza K, Yates B, Triffault S, Gangar S, Long B, et al. Strategy to detect pre-existing immunity to AAV gene therapy. Gene Ther. 2017 Dec;24(12):768–78. doi:10.1038/gt.2017.95 PubMed PMID: 29106404; PubMed Central PMCID: PMC5746592.

21.	Aronson SJ, Veron P, Collaud F, Hubert A, Delahais V, Honnet G, et al. Prevalence and Relevance of Pre-Existing Anti-Adeno-Associated Virus Immunity in the Context of Gene Therapy for Crigler-Najjar Syndrome. Hum Gene Ther. 2019 Oct;30(10):1297–305. doi:10.1089/hum.2019.143 PubMed PMID: 31502485; PubMed Central PMCID: PMC6763963.

22.	Leborgne C, Latournerie V, Boutin S, Desgue D, Quéré A, Pignot E, et al. Prevalence and long-term monitoring of humoral immunity against adeno-associated virus in Duchenne Muscular Dystrophy patients. Cell Immunol. 2019 Aug;342:103780. doi:10.1016/j.cellimm.2018.03.004 PubMed PMID: 29571923.

23.	Andrzejewski S, Murali A, Ramlogan-Steel C, Edwards KP, Efron N, Steel JC, et al. Adeno-associated virus neutralising antibodies in type 1 diabetes mellitus. Gene Ther. 2019 Jun;26(6):250–63. doi:10.1038/s41434-019-0076-5 PubMed PMID: 30962537.

24.	Stanford S, Pink R, Creagh D, Clark A, Lowe G, Curry N, et al. Adenovirus-associated antibodies in UK cohort of hemophilia patients: A seroprevalence study of the presence of adenovirus-associated virus vector-serotypes AAV5 and AAV8 neutralizing activity and antibodies in patients with hemophilia A. Res Pract Thromb Haemost. 2019 Apr;3(2):261–7. doi:10.1002/rth2.12177 PubMed PMID: 31011710; PubMed Central PMCID: PMC6462753.

25.	Perocheau DP, Cunningham S, Lee J, Antinao Diaz J, Waddington SN, Gilmour K, et al. Age-Related Seroprevalence of Antibodies Against AAV-LK03 in a UK Population Cohort. Hum Gene Ther. 2019 Jan;30(1):79–87. doi:10.1089/hum.2018.098 PubMed PMID: 30027761; PubMed Central PMCID: PMC6343184.

26.	Kruzik A, Koppensteiner H, Fetahagic D, Hartlieb B, Dorn S, Romeder-Finger S, et al. Detection of Biologically Relevant Low-Titer Neutralizing Antibodies Against Adeno-Associated Virus Require Sensitive In Vitro Assays. Hum Gene Ther Methods. 2019 Apr;30(2):35–43. doi:10.1089/hgtb.2018.263 PubMed PMID: 30734588.

27.	Kruzik A, Fetahagic D, Hartlieb B, Dorn S, Koppensteiner H, Horling FM, et al. Prevalence of Anti-Adeno-Associated Virus Immune Responses in International Cohorts of Healthy Donors. Mol Ther Methods Clin Dev. 2019 Sep 13;14:126–33. doi:10.1016/j.omtm.2019.05.014 PubMed PMID: 31338384; PubMed Central PMCID: PMC6629972.

28.	Lee S, Kang IK, Kim JH, Jung BK, Park K, Chang H, et al. Relationship Between Neutralizing Antibodies Against Adeno-Associated Virus in the Vitreous and Serum: Effects on Retinal Gene Therapy. Translational Vision Science & Technology. 2019 Apr 9;8(2):14. doi:10.1167/tvst.8.2.14

29.	Shao W, Pei X, Cui C, Askew C, Dobbins A, Chen X, et al. Superior human hepatocyte transduction with Adeno-associated Virus Vector Serotype 7. Gene Ther. 2019 Dec;26(12):504–14. doi:10.1038/s41434-019-0104-5 PubMed PMID: 31570819; PubMed Central PMCID: PMC6923567.

30.	Andrzejewski S, Moyle PM, Stringer BW, Steel JC, Layton CJ. Neutralisation of adeno-associated virus transduction by human vitreous humour. Gene Ther. 2021 May;28(5):242–55. doi:10.1038/s41434-020-0162-8

31.	Daniel HDJ, Kumar S, Kannangai R, Lakshmi KM, Agbandje-Mckenna M, Coleman K, et al. Prevalence of Adeno-Associated Virus 3 Capsid Binding and Neutralizing Antibodies in Healthy and Hemophilia B Individuals from India. Hum Gene Ther. 2021 May;32(9–10):451–7. doi:10.1089/hum.2020.258 PubMed PMID: 33207962.

32.	Gehrke M, Diedrichs-Möhring M, Bogedein J, Büning H, Michalakis S, Wildner G. Immunogenicity of Novel AAV Capsids for Retinal Gene Therapy. Cells. 2022 Jun 9;11(12):1881. doi:10.3390/cells11121881 PubMed PMID: 35741009; PubMed Central PMCID: PMC9221425.

33.	Haar J, Blazevic D, Strobel B, Kreuz S, Michelfelder S. MSD-based assays facilitate a rapid and quantitative serostatus profiling for the presence of anti-AAV antibodies. Mol Ther Methods Clin Dev. 2022 Jun 9;25:360–9. doi:10.1016/j.omtm.2022.04.008 PubMed PMID: 35573045; PubMed Central PMCID: PMC9065051.

34.	Kashiwakura Y, Baatartsogt N, Yamazaki S, Nagao A, Amano K, Suzuki N, et al. The seroprevalence of neutralizing antibodies against the adeno-associated virus capsids in Japanese hemophiliacs. Mol Ther Methods Clin Dev. 2022 Dec 8;27:404–14. doi:10.1016/j.omtm.2022.10.014 PubMed PMID: 36381300; PubMed Central PMCID: PMC9661668.

35.	Boyce S, James I, Rangarajan S, Curry N, Bagot C, Austin S, et al. Seroprevalence to adeno-associated virus type 6 in people with hemophilia B from a UK adult cohort. Res Pract Thromb Haemost. 2022 May;6(4):e12705. doi:10.1002/rth2.12705 PubMed PMID: 35677030; PubMed Central PMCID: PMC9166283.

36.	Chandler RJ, Di Pasquale G, Sloan JL, McCoy S, Hubbard BT, Kilts TM, et al. Systemic gene therapy for methylmalonic acidemia using the novel adeno-associated viral vector 44.9. Mol Ther Methods Clin Dev. 2022 Dec 8;27:61–72. doi:10.1016/j.omtm.2022.09.001 PubMed PMID: 36186952; PubMed Central PMCID: PMC9490190.

37.	Daniel HDJ, Kumar S, Kannangai R, J F, Joel JN, Abraham A, et al. Age-stratified adeno-associated virus serotype 3 neutralizing and total antibody prevalence in hemophilia A patients from India. J Med Virol. 2022 Sep;94(9):4542–7. doi:10.1002/jmv.27859 PubMed PMID: 35577570.

38.	Wang Y, Yang C, Hu H, Chen C, Yan M, Ling F, et al. Directed evolution of adeno-associated virus 5 capsid enables specific liver tropism. Mol Ther Nucleic Acids. 2022 Jun 14;28:293–306. doi:10.1016/j.omtn.2022.03.017 PubMed PMID: 35474733; PubMed Central PMCID: PMC9010518.

39.	Sierra-Delgado JA, Likhite S, Bautista PK, Gómez-Ochoa SA, Echeverría LE, Guío E, et al. Prevalence of Neutralizing Antibodies against Adeno-Associated Virus Serotypes 1, 2, and 9 in Non-Injected Latin American Patients with Heart Failure-ANVIAS Study. Int J Mol Sci. 2023 Mar 14;24(6):5579. doi:10.3390/ijms24065579 PubMed PMID: 36982654; PubMed Central PMCID: PMC10051173.

40.	Hsi J, Mietzsch M, Chipman P, Afione S, Zeher A, Huang R, et al. Structural and antigenic characterization of the avian adeno-associated virus capsid. J Virol. 2023 Oct 31;97(10):e0078023. doi:10.1128/jvi.00780-23 PubMed PMID: 37702486; PubMed Central PMCID: PMC10617571.

41.	Abdul TY, Hawse GP, Smith J, Sellon JL, Abdel MP, Wells JW, et al. Prevalence of AAV2.5 neutralizing antibodies in synovial fluid and serum of patients with osteoarthritis. Gene Ther. 2023 Aug;30(7–8):587–91. doi:10.1038/s41434-022-00326-5 PubMed PMID: 35260797; PubMed Central PMCID: PMC9598672.

42.	Verma S, Nwosu SN, Razdan R, Upadhyayula SR, Phan HC, Koroma AA, et al. Seroprevalence of Adeno-Associated Virus Neutralizing Antibodies in Males with Duchenne Muscular Dystrophy. Human Gene Therapy. 2023 May;34(9–10):430–8. doi:10.1089/hum.2022.081

43.	Chhabra A, Bashirians G, Petropoulos CJ, Wrin T, Paliwal Y, Henstock PV, et al. Global seroprevalence of neutralizing antibodies against adeno-associated virus serotypes used for human gene therapies. Mol Ther Methods Clin Dev. 2024 Sep 12;32(3):101273. doi:10.1016/j.omtm.2024.101273 PubMed PMID: 39022744; PubMed Central PMCID: PMC11253686.

44.	Pabinger I, Ayash-Rashkovsky M, Escobar M, Konkle BA, Mingot-Castellano ME, Mullins ES, et al. Multicenter assessment and longitudinal study of the prevalence of antibodies and related adaptive immune responses to AAV in adult males with hemophilia. Gene Ther. 2024 May;31(5):273–84. doi:10.1038/s41434-024-00441-5

45.	Wei C, Li D, Zhang M, Zhao Y, Liu Y, Fan Y, et al. Prevalence of Adeno-Associated Virus-9-Neutralizing Antibody in Chinese Patients with Duchenne Muscular Dystrophy. Hum Gene Ther. 2024 Jan;35(1–2):26–35. doi:10.1089/hum.2023.117 PubMed PMID: 38084965.

46.	Qin X, Li H, Zhao H, Xiang K, Liu S, Lou R, et al. Prevalence of Neutralizing Antibodies Against AAV Serotypes 2 and 9 in Healthy Participants from Multiple Centers Across China and Patients with DMD/BMD. Hum Gene Ther. 2024 Dec;35(23–24):969–77. doi:10.1089/hum.2024.079 PubMed PMID: 39607725; PubMed Central PMCID: PMC11659434.

47.	Mylvara AV, Davidson CD, Alexander D, Venditti CP, Porter FD. Prevalence of Neutralizing Antibodies to AAV2 and AAV9 in Individuals with Niemann-Pick Disease, Type C1. Human Gene Therapy. 2025 Apr;36(7–8):729–37. doi:10.1089/hum.2024.233

48.	Zhao Q, Yu S, Fu D, Wu Z, Zhou J, Yang Y, et al. Pre-existing Anti-AAV9 antibodies in the Chinese healthy and rare disease populations: Implications for gene therapy. Virus Research. 2025 Apr 1;354:199549. doi:10.1016/j.virusres.2025.199549

49.	Klamroth R, Recht M, Key NS, Miesbach W, Pipe SW, Kaczmarek R, et al. Natural history of preexisting AAV5 antibodies in adults with hemophilia B during the lead-in of the etranacogene dezaparvovec phase 3 study. Molecular Therapy Methods & Clinical Development. 2025 Sep 11;33(3):101568. doi:10.1016/j.omtm.2025.101568

50.	Parra R, Bosch A, Sánchez A, Caparros C, Kim HK, Lwoff N. Seroprevalence of anti-AAV antibodies in a healthy adult Spanish population: findings from the SAAVIA study. Gene Ther. 2026 Mar;33(2):156–63. doi:10.1038/s41434-026-00597-2 PubMed PMID: 41820593.
